# The ARID1B spectrum in 143 patients: from nonsyndromic intellectual disability to Coffin–Siris syndrome

**DOI:** 10.1038/s41436-018-0330-z

**Published:** 2018-11-08

**Authors:** Pleuntje J. van der Sluijs, Sandra Jansen, Samantha A. Vergano, Miho Adachi-Fukuda, Yasemin Alanay, Adila AlKindy, Anwar Baban, Allan Bayat, Stefanie Beck-Wödl, Katherine Berry, Emilia K. Bijlsma, Levinus A. Bok, Alwin F. J. Brouwer, Ineke van der Burgt, Philippe M. Campeau, Natalie Canham, Krystyna Chrzanowska, Yoyo W. Y. Chu, Brain H. Y. Chung, Karin Dahan, Marjan De Rademaeker, Anne Destree, Tracy Dudding-Byth, Rachel Earl, Nursel Elcioglu, Ellen R. Elias, Christina Fagerberg, Alice Gardham, Blanca Gener, Erica H. Gerkes, Ute Grasshoff, Arie van Haeringen, Karin R. Heitink, Johanna C. Herkert, Nicolette S. den Hollander, Denise Horn, David Hunt, Sarina G. Kant, Mitsuhiro Kato, Hülya Kayserili, Rogier Kersseboom, Esra Kilic, Malgorzata Krajewska-Walasek, Kylin Lammers, Lone W. Laulund, Damien Lederer, Melissa Lees, Vanesa López-González, Saskia Maas, Grazia M. S. Mancini, Carlo Marcelis, Francisco Martinez, Isabelle Maystadt, Marianne McGuire, Shane McKee, Sarju Mehta, Kay Metcalfe, Jeff Milunsky, Seiji Mizuno, John B. Moeschler, Christian Netzer, Charlotte W. Ockeloen, Barbara Oehl-Jaschkowitz, Nobuhiko Okamoto, Sharon N. M. Olminkhof, Carmen Orellana, Laurent Pasquier, Caroline Pottinger, Vera Riehmer, Stephen P. Robertson, Maian Roifman, Caroline Rooryck, Fabienne G. Ropers, Monica Rosello, Claudia A. L. Ruivenkamp, Mahmut S. Sagiroglu, Suzanne C. E. H. Sallevelt, Amparo Sanchis Calvo, Pelin O. Simsek-Kiper, Gabriela Soares, Lucia Solaeche, Fatma Mujgan Sonmez, Miranda Splitt, Duco Steenbeek, Alexander P. A. Stegmann, Constance T. R. M. Stumpel, Saori Tanabe, Eyyup Uctepe, G. Eda Utine, Hermine E. Veenstra-Knol, Sunita Venkateswaran, Catheline Vilain, Catherine Vincent-Delorme, Anneke T. Vulto-van Silfhout, Patricia Wheeler, Golder N. Wilson, Louise C. Wilson, Bernd Wollnik, Tomoki Kosho, Dagmar Wieczorek, Evan Eichler, Rolph Pfundt, Bert B. A. de Vries, Jill Clayton-Smith, Gijs W. E. Santen

**Affiliations:** 1 Department of Clinical Genetics, Leiden University Medical Center, Leiden, The Netherlands; 2 Department of Human Genetics, Donders Institute for Brain, Cognition and Behaviour, Radboud University Medical Center, Nijmegen, The Netherlands; 3 Division of Medical Genetics and Metabolism, Children’s Hospital of the King’s Daughters, Norfolk, VA USA; 4 Department of Pediatrics, St. Marianna University School of Medicine, Kanagawa, Japan; 5 School of Medicine, Department of Pediatrics, Pediatric Genetics Unit, Acibadem University, Istanbul, Turkey; 6 Department of Genetics, Sultan Qaboos University Hospital, Muscat, Oman; 7 Pediatric Cardiology and Cardiac Surgery Department, Bambino Gesù Children Hospital and Research Institute, IRCCS, Rome, Italy; 8 Copenhagen University Hospital Hvidovre, Copenhagen, Denmark; 9 Department of Molecular Genetics and Applied Genomics, University Hospital Tübingen, Tübingen, Germany; 10 Department of Medical Genetics, Shodair Hospital, Helena, MT USA; 11 Department of Pediatrics, Màxima Medical Centre, Veldhoven, The Netherlands; 12 Department of Paediatrics, Nij Smellinghe Hospital, Drachten, The Netherlands; 13 Department of Human Genetics, Radboud University Medical Center, Nijmegen, The Netherlands; 14 Department of Pediatrics, CHU Sainte-Justine and University of Montreal, Montreal, QC Canada; 15 North West Thames Regional Genetics Service, Northwick Park Hospital, Harrow, United Kingdom; 16 Cheshire and Merseyside Regional Genetics Service, Liverpool Women’s Hospital, Crown Street, Liverpool, United Kingdom; 17 Department of Medical Genetics, The Children’s Memorial Health Institute, Warsaw, Poland; 18 Department of Paediatrics and Adolescent Medicine, Li Ka Shing Faculty of Medicine, The University of Hong Kong, Hong Kong, SAR China; 19 Center for Human Genetics, Institute of Pathology and Genetics, Gosselies, Belgium; 20 Center for Medical Genetics, Vrije Universiteit Brussels, Brussels, Belgium; 21 Hunter Genetics and University of Newcastle, GrowUpWell Priority Research Centre, Newcastle, Australia; 22 Department of Psychiatry and Behavioral Sciences, University of Washington, Seattle, WA USA; 23 Department of Pediatric Genetics, Marmara University Pendik Hospital, Istanbul, Turkey; 24 Department of Pediatrics and Genetics, University of Colorado Denver School of Medicine, Aurora, CO USA; 25 Department of Clinical Genetics, Odense University Hospital, Odense, Denmark; 26 Department of Genetics, Cruces University Hospital, Biocruces Health Research Institute, Vizcayam, Spain; 27 University of Groningen, University Medical Center Groningen, Department of Genetics, Groningen, The Netherlands; 28 Department of Rehabilitation Medicine, Leiden University Medical Center, Leiden, The Netherlands; 29 Institute for Medical Genetics and Human Genetics, Charité Universitätsmedizin, Berlin, Germany; 30 Wessex Clinical Genetics Service, Princess Anne Hospital, Southampton, United Kingdom; 31 Department of Pediatrics, Showa University School of Medicine, Tokyo, Japan; 32 Medical Genetics Department, Koç University School of Medicine (KUSoM), İstanbul, Turkey; 33 Department of Clinical Genetics, Sophia Children’s Hospital, Erasmus MC, Rotterdam, The Netherlands; 34 Department of Pediatric Genetics, Hematology Oncology Research & Training Children’s Hospital, Ankara, Turkey; 35 Department of Medical Genetics, Dayton Children’s Hospital, Dayton, OH USA; 36 Department of Paediatrics, Odense University Hospital, Odense, Denmark; 37 Department of Clinical Genetics, Great Ormond Street Hospital NHS Foundation Trust, London, United Kingdom; 38 Sección de Genética Médica, Servicio de Pediatria, Hospital Clinico Universitario Virgen de la Arrixaca, IMIB-Arrixaca, CIBERER-ISCIII, Murcia, Spain; 39 Department of Clinical Genetics, Academic Medical Center, University of Amsterdam, Amsterdam, The Netherlands; 40 Unidad de Genética, Hospital Universitario y Politécnico La Fe, Valencia, Spain; 41 Department of Molecular and Human Genetics, Baylor College of Medicine, One Baylor Plaza, Houston, TX USA; 42 Northern Ireland Regional Genetics Centre, Belfast City Hospital, Belfast, Ireland; 43 East Anglian Regional Genetics Service, Cambridge University Hospitals NHS Foundation Trust, Addenbrooke’s Hospital, Cambridge, United Kingdom; 44 Manchester Centre for Genomic Medicine, Division of Evolution and Genomic Sciences, St Mary’s Hospital, Manchester University Hospitals NHS Foundation Trust Manchester Academic Health Sciences Centre, Manchester, United Kingdom; 45 Center for Human Genetics Inc, Cambridge, MA USA; 46 Department of Pediatrics, Central Hospital, Aichi Human Service Center, Kasugai, Aichi Japan; 47 Department of Pediatrics, Geisel School of Medicine, Dartmouth College, Hanover, NH USA; 48 Institute of Human Genetics, University Hospital of Cologne, Cologne, Germany; 49 Gemeinschaftspraxis für Humangenetik Homburg/Saar, Homburg, Germany; 50 Department of Medical Genetics, Osaka Women’s and Children’s Hospital, Osaka, Japan; 51 Willem Alexander Children’s Hospital, Leiden University Medical Center, Leiden, The Netherlands; 52 CRMR Déficiences intellectuelles, Service de Génétique Médicale, CLAD Ouest CHU Hôpital Sud, Rennes, France; 53 All Wales Medical Genetics Service, Glan Clwyd Hospital, Rhyl, United Kingdom; 54 Dunedin School of Medicine, University of Otago, Dunedin, New Zealand; 55 Division of Clinical and Metabolic Genetics, Department of Paediatrics, The Hospital for Sick Children, University of Toronto, Toronto, ON Canada; 56 The Prenatal Diagnosis and Medical Genetics Program, Department of Obstetrics and Gynecology, Mount Sinai Hospital, Toronto, ON Canada; 57 Department of Medical Genetics, CHU Bordeaux, Bordeaux, France; 58 Department of Pediatrics, Leiden University Medical Center, Leiden, The Netherlands; 59 Genpute Computation Technologies Company, Istanbul, Turkey; 60 Department of Clinical Genetics and GROW-School for Oncology and Developmental Biology, Maastricht University Medical Center, Maastricht, The Netherlands; 61 Servicio de Pediatría, Hospital Universitario Doctor Peset, Valencia, Spain; 62 Department of Pediatric Genetics, Ihsan Dogramaci Children’s Hospital, Hacettepe University School of Medicine, Ankara, Turkey; 63 Jacinto de Magalhães Medical Genetics Center, Centro Hospitalar do Porto, Porto, Portugal; 64 Departamento de neurometabólicas, Hospital Universitario Son Espases, Palma de Mallorca, Spain; 65 Karadeniz Technical University, Faculty of Medicine, Dept of Child Neurology, Retired Professor, Trabzon, Turkey; 66 Northern Genetics Service, Institute of Genetics Medicine, Newcastle upon Tyne, United Kingdom; 67 Division of Pediatrics, Yamagata Prefectural and Sakata Munici pal Hospital Organization Nihon-Kai General Hospital, Sakata, Japan; 68 Enva Engineering, Ankara, Turkey; 69 Division of Neurology, Department of Pediatrics, Children’s Hospital of Eastern Ontario, University of Ottawa, Ottawa, ON Canada; 70 Department of Genetics, Hôpital Universitaire des Enfants Reine Fabiola, ULB Center of Medical Genetics, Université Libre de Bruxelles, Brussels, Belgium; 71 Department of Genetics, Hôpital Erasme. ULB Center of Medical Genetics, Université Libre de Bruxelles, Brussels, Belgium; 72 Service de génétique clinique Guy Fontaine, CHRU de Lille–Hôpital Jeanne de Flandre, Lille, France; 73 Division of Genetics, Arnold Palmer Hospital, Orlando, FL USA; 74 KinderGenome Genetics, Medical City Hospital Dallas, Dallas, TX USA; 75 Institute of Human Genetics, University Medical Center Göttingen, Göttingen, Germany; 76 Center for Medical Genetics, Shinshu University Hospital, Matsumoto, Japan; 77 Institute of Human Genetics, Medical Faculty, Heinrich-Heine-University, Düsseldorf, Germany; 78 Department of Genome Sciences, University of Washington School of Medicine, Seattle, WA USA

**Keywords:** ARID1B, Coffin–Siris syndrome, intellectual disability, bias

## Abstract

**Purpose:**

Pathogenic variants in ARID1B are one of the most frequent causes of intellectual disability (ID) as determined by large-scale exome sequencing studies. Most studies published thus far describe clinically diagnosed Coffin–Siris patients (ARID1B-CSS) and it is unclear whether these data are representative for patients identified through sequencing of unbiased ID cohorts (ARID1B-ID). We therefore sought to determine genotypic and phenotypic differences between ARID1B-ID and ARID1B-CSS. In parallel, we investigated the effect of different methods of phenotype reporting.

**Methods:**

Clinicians entered clinical data in an extensive web-based survey.

**Results:**

79 ARID1B-CSS and 64 ARID1B-ID patients were included. CSS-associated dysmorphic features, such as thick eyebrows, long eyelashes, thick alae nasi, long and/or broad philtrum, small nails and small or absent fifth distal phalanx and hypertrichosis, were observed significantly more often (*p* < 0.001) in ARID1B-CSS patients. No other significant differences were identified.

**Conclusion:**

There are only minor differences between ARID1B-ID and ARID1B-CSS patients. ARID1B-related disorders seem to consist of a spectrum, and patients should be managed similarly. We demonstrated that data collection methods without an explicit option to report the absence of a feature (such as most Human Phenotype Ontology-based methods) tended to underestimate gene-related features.

## INTRODUCTION

The overall prevalence of intellectual disability (ID) has been estimated at around 1%. Given the increasing number of genes involved in ID, exome sequencing is becoming the first method of choice to identify the underlying genetic cause in patients with ID.^1^ This unbiased approach detects clearly pathogenic variants in patients without the typically associated phenotype, indicating that variability in expression is higher than previously thought, confirming the existence of ascertainment bias. This bias may be mitigated by establishing the frequency of cardinal features in patients diagnosed through sequencing of unselected populations, although selection criteria for patients who undergo sequencing still cause a degree of bias. However, studies in unbiased populations suffer from another, less appreciated bias: they may *under*estimate the frequency of cardinal features because of the way that data are collected in large research studies. We would like to coin this phenomenon “phenotype underreporting bias”: typically, a busy clinician is requested to supply several Human Phenotype Ontology (HPO) terms, and there is no guarantee that all features have been assessed or that all the clinical information is reported by the clinician. Consequently, it is not possible to make a distinction between the absence of a feature and unknown status, especially when specific diagnostic procedures are required to assess a feature (e.g., a magnetic resonance image [MRI] scan for agenesis of the corpus callosum [ACC]).

The ARID1B phenotype represents a good case study to investigate these biases, because the associated phenotypes range from clearly recognizable Coffin–Siris syndrome (ARID1B-CSS) to less specific ID^2,3^ (ARID1B-ID). *ARID1B* is by far the most frequently mutated gene (51–75%) (refs. ^4–6^) in Coffin Siris syndrome (CSS) (OMIM 135900) and large-scale exome sequencing studies invariably find that pathogenic variants in ARID1B are among the most frequently identified causes in unspecified ID cohorts (usually around 1%) (refs. ^1,2^).

CSS is characterized by “developmental or cognitive delay, hypotonia, sparse scalp hair, distinctive facial features, aplasia or hypoplasia of the distal phalanx or nail of the fifth and additional digits, and hypertrichosis.”^7^ Approximately 70 ARID1B-CSS patients^4–6,8–12^ have been described. Roughly 30 ARID1B-ID patients have been described in some detail^2,13–24^ and an additional 23 patients with pathogenic variants in ARID1B were identified in genome-wide research studies^1,24^ where detailed clinical information is generally unavailable. Data on the frequency of typical CSS features in the ARID1B-ID population are lacking because a substantial number of these patients were published before the link between ARID1B and CSS was known. For example, in Hoyer et al.^2^ ACC was not specifically reported, although this is now known as a frequent feature in ARID1B-CSS.^11^ Therefore, the precise prevalence of these features cannot be estimated. The availability of unbiased information is crucial now that exome sequencing is being performed increasingly in neonatal and prenatal settings, and a reliable prognosis can only be given based on unbiased data.

The first aim of this study was therefore to overcome ascertainment and phenotype underreporting biases, by acquiring detailed clinical data of a large cohort of ARID1B-ID patients. The second aim was to determine whether the frequencies of features differ between ARID1B-CSS and ARID1B-ID patients, as would be expected at least for typical CSS features.

## MATERIALS AND METHODS

### Patient ascertainment

We developed a web-based survey (www.arid1bgene.com) based on previously reported features of ARID1B patients. This website is part of the Human Disease Genes website series (HDG), a collection of websites aimed at informing professionals about genes and copy-number variations and their clinical consequences (http://humandiseasegenes.com/). The survey was open to all clinicians of patients with pathogenic variants in *ARID1B*. Data were contributed by pediatric neurologists, pediatricians, and in most instances by clinical geneticists. Some clinicians contacted us, others were approached based on publications, conference presentations, submissions to databases like DECIPHER, or through large laboratories. We also included patients from our previous studies^4–6,9,11^ and those referred to our national CSS expertise center in Leiden, The Netherlands.

The institutional review board of the Leiden University Medical Center, Leiden, The Netherlands provided an approval waiver for this study.

### Data assessment

When only partial variant data was given, the remaining information was recovered using Alamut version 2.6.0. When the standard deviation score (SDS) was not reported, but raw data on weight, height, or occipital–frontal circumference (OFC) was available, the SDS was determined using published growth charts.^25^

After initial analyses we recontacted contributing clinicians by email to inquire about features frequently reported in open-ended questions.

We used a nominal *p* value of 0.05 as a cut-off for significance. However, given that we assessed 90 features, multiplicity correction by Bonferroni suggests that *p* values above 0.0006 (0.05/90) should be treated with care. All analyses were executed using SPSS version 23. R version 3.4.1 was used to create graphs, including the *survival* package (version 2.41-3).

## RESULTS

Data from 143 individuals with pathogenic variants in *ARID1B* were included in the database. We received additional data regarding features recurrently indicated in our open-ended questions (Table 1 features marked with “++”, Supplementary Table S4) of 95 patients. Supplementary Figure S1 displays facial photographs and hands or feet from two ARID1B-ID and two ARID1B-CSS patients. Parents provided consent for publication of these pictures.

**Table 1:** Clinical characteristics of ARID1B patients

	Total		ARID1B-CSS		ARID1B-ID			
Clinical features^d^	*n*= 143	%	*n =*79	%	*n =*64	%	*p* value	Test
Sex (female)	143	48.3	79	57.0	64	37.5	0.028	^a^
**Growth parameters & development**								
Gestational age, weeks (mean; SD)	133	39.0; 2.1	75	39.1; 2.0	58	38.9; 2.4	0.879	^b^
Birthweight (<–2 SDS)	129	5.4	74	6.8	55	3.6	0.506	^c^
Height at birth (<–2 SDS)	43	9.3	27	11.1	16	6.3	0.660	^c^
OFC at birth (<–2 SDS)	51	3.9	35	2.9	16	6.3	0.232	^c^
Age last measurements, years (median; min–max)	143	10; 0–51	79	10; 0–36	64	9; 0.5–51	0.682	^b^
Weight (<–2 SDS)	92	6.5	46	8.7	46	4.3	0.571	^c^
Height (<–2 SDS)	122	30.3	70	37.1	52	21.2	0.177	^c^
OFC (<–2 SDS)	105	2.9	63	3.2	42	2.4	0.670	^c^
Motor skills gross, delayed	103	99.0	46	97.8	57	100.0	0.447	^c^
Motor skills fine, delayed	100	95.0	44	97.7	56	92.9	0.381	^c^
Speech, delayed	131	65.6	75	68.0	56	62.5	0.106	^c^
Obstructive sleep apnea^e^	71	8.5	34	0.0	37	16.2	0.026	^c^
Laryngomalacia^e^	90	19.8	47	17.0	44	22.7	0.466	^a^
Feeding difficulties	121	69.4	62	62.9	59	76.3	0.111	^a^
Start of feeding difficulties	71		34		37		0.345	^c^
Birth		76.1		76.5		75.7		
Before 6 months		16.9		20.6		13.5		
After 6 months		7.0		2.9		10.8		
Duration of feeding problems	58		23		35		0.639	^c^
Brief		46.6		39.1		51.4		
Several years		6.9		8.7		5.7		
Ongoing		46.6		52.2		42.9		
Tube feeding	65	16.9	22	13.6	43	18.6	0.409	^c^
0–6 months		10.8		4.5		14.0		
6–12 months		3.1		4.5		2.3		
1–3 years		1.5		0.0		2.3		
Recurrent infections	75	57.3	30	63.3	45	53.3	0.391	^a^
Upper airway tract		17.3		10.0		22.2		
Lower airway tract		2.7		0.0		4.4		
ENT infections		12.0		10.0		13.3		
Otitis media		14.7		13.3		15.6		
Urinary tract		2.7		3.3		2.2		
**Neurological features**								
Intellectual disability	127	99.2	70	98.6	57	100.0	0.015	^c^
Normal–mild		3.1		1.4		5.3		
Mild		28.3		38.6		15.8		
Mild–moderate		15.7		8.6		24.6		
Moderate		22.0		22.9		21.1		
Moderate–severe		16.5		17.1		15.8		
Severe		13.4		10.0		17.5		
Hypotonia	116	81.0	71	80.3	45	82.2	0.795	^a^
Seizures	142	27.5	78	28.2	64	26.6	0.880	^c^
No seizures, but abnormal EEG		5.6		6.4		4.7		
Seizure frequency	18		9		9		0.671	^c^
Once		27.8		11.1		44.4		
Less than once a year		11.1		22.2		0.0		
Once a year		33.3		44.4		22.2		
Once a month		11.1		11.1		11.1		
1/2 a month		5.6		0.0		11.1		
≥2 per month		5.6		0.0		11.1		
Agenesis of the corpus callosum	101	28.7	62	29.0	39	28.2	0.344	^c^
Partial/hypoplasia		13.9		17.7		7.7		
Neuroradiology	47	87.2	17	94.1	30	83.3	0.305	^a^
Delayed myelination		17.0		11.8		20.0		
Mega cisterna magna		14.9		23.5		10.0		
Colpocephaly		10.6		11.8		10.0		
Hypoplasia		4.3		0.0		6.7		
Enlarged Virchow–Robin spaces		4.3		5.9		3.3		
**Vision and hearing impairments**								
Vision impaired	109	48.6	62	45.2	47	53.2	0.406	^a^
Vision problems	68	70.6	33	78.8	35	62.9	0.320	^c^
Astigmatism		16.2		24.2		8.6		
Strabismus		30.9		36.4		25.7		
Optic nerve hypoplasia		2.9		6.1		0.0		
Nystagmus		8.8		6.1		11.4		
Refraction error		10.3		9.1		11.4		
Myopia	102	27.5	59	18.6	43	39.5	0.020	^a^
Hypermetropia	50	18.0	21	28.6	29	10.3	0.140	^c^
Abnormal eye exam	40	17.5	15	6.7	25	24.0	0.224	^c^
Hearing loss	122	22.1	71	18.3	51	27.5	0.157	^a^
Hearing loss, conductive		6.6		1.4		13.7		
Hearing loss, bilateral		11.5		8.5		15.7		
Hearing loss, unilateral		4.9		5.6		3.9		
Eartubes		4.9		4.2		5.9		
Start hearing problems, congenital	11	63.6	3	66.7	8	62.5	0.109	^c^
Hearing aid	5	80.0	2	100.0	3	66.7	0.665	^c^
**Dysmorphic features**								
Coarse face	121	81.8	62	90.3	59	72.9	0.013	^a^
Hairline (low anterior and/or posterior)	91	69.2	45	75.6	46	63.0	0.196	^a^
Scalp hair, abnormal	129	79.1	78	83.3	51	72.5	0.141	^a^
Sparse		58.1		62.8		51.0		
Forehead (broad or narrow)	95	42.1	49	28.6	46	56.5	0.000	^c^
Broad		22.1		6.1		39.1		
Narrow		20.0		22.4		17.4		
Eyelashes, long	131	63.4	79	75.9	52	44.2	0.000	^a^
Eyebrows, thick	134	81.3	78	91.0	56	67.9	0.001	^a^
Ptosis	133	20.3	77	20.8	56	19.6	0.872	^a^
Tear duct nonfunctioning or absent	93	15.1	54	16.7	39	12.8	0.609	^a^
Nasal bridge, abnormal	100	61.0	59	62.7	41	58.5	0.050	^a^
Wide		34.0		40.7		24.4		
Flat		21.0		20.3		22.0		
Broad		12.0		5.1		22.0		
Nasal tip, abnormal	129		76		53		0.002	^a^
Broad		58.1		61.8		52.8		
Upturned (anteverted nares)		29.5		39.5		15.1		
Nose, abnormal	83	47.0	43	53.5	40	40.0	0.022	^a^
Short		26.5		39.5		12.5		
Long		20.5		14.0		27.5		
Alae nasi, thick	107	55.1	69	66.7	38	34.2	0.001	^a^
Nasal base, broad	88	48.9	48	43.8	40	55.0	0.392	^a^
Philtrum, abnormal	109	78.9	72	86.1	37	64.9	0.001	^a^
Short		24.8		29.2		16.2		
Long		44.0		48.6		35.1		
Broad		34.9		44.4		16.2		
Mouth, large	131	68.7	75	76.0	56	58.9	0.037	^a^
Upper vermillion, abnormal	127	56.7	75	60.0	52	51.9	0.366	^a^
Thin		35.4		45.3		21.2		
Thick		21.3		14.7		30.8		
Lower vermillion, thick	125	69.6	76	78.9	49	55.1	0.005	^a^
Lower lip, drooping	71	56.3	30	76.7	41	41.5	0.004	^a^
Cleft palate/submucous cleft	90	6.7	35	14.3	55	1.8	0.031	^a^
Cleft palate		2.2		5.7		0.0		
Bifid uvula		2.2		5.7		0.0		
Submucous cleft		3.3		5.7		1.8		
High arched palate	85	16.5	31	22.6	54	13.0	0.250	^a^
Ears, abnormal	122	52.5	66	57.6	56	46.4	0.433	^a^
Low-set		9.8		13.6		5.4		
Posterior rotated		7.4		9.1		5.4		
Hypertrichosis	128	86.7	76	94.7	52	75.0	0.001	^a^
**Musculoskeletal anomalies**								
Scoliosis	123	26.0	70	27.1	53	24.5	0.743	^a^
Pectus, excavatum	104	13.5	57	14.0	47	12.8	0.850	^a^
Primary dentition, delayed	65	44.6	40	50.0	25	36.0	0.313	^a^
Permanent dentition, delayed	33	48.5	18	33.3	15	66.7	0.056	^a^
Widely spaced teeth	72	41.7	40	40.0	32	43.8	0.748	^a^
Bone age, delayed	40	47.5	30	46.7	10	50.0	1.000	^c^
Joint laxity	88	60.2	52	61.5	36	58.3	0.763	^a^
Early arthritis	75	5.3	36	5.6	39	5.1	1.000	^c^
Clinodactyly	77	36.4	42	45.2	35	25.7	0.076	^a^
Short phalanges	49	34.7	34	41.2	15	20.0	0.151	^a^
Complete absent or small 5th distal phalanx	110	40.0	66	60.6	44	9.1	0.000	^c^
Prominent distal phalanges;	102	24.5	64	31.3	38	13.2	0.040	^a^
Prominent interphalangeal joints	103	21.4	64	28.1	39	10.3	0.032	^a^
Brachydactyly general	60	16.7	19	15.8	41	17.1	1.000	^c^
Brachydactyly fifth finger	68	30.9	22	50.0	46	21.7	0.018	^a^
Small nails	122	54.9	73	68.5	49	34.7	0.000	^a^
Which nails, 5th finger and/or toe	106	55.7	67	65.7	39	38.5	0.007	^a^
Which nails, all	53	11.3	29	20.7	24	0.0	0.027	^c^
Fetal finger pads	100	29.0	50	26.0	50	32.0	0.509	^a^
**Intestinal**								
Inguinal hernia	90	7.8	46	2.2	44	13.6	0.056	^c^
Intestinal problems	105	48.6	60	36.7	45	64.4	0.000	^a^
Constipation		30.5		21.7		42.2		
Gastroesophageal reflux		17.1		13.3		22.2		
Diarrhea		4.8		3.3		6.7		
Pyloric Stenosis		2.9		5.0		0.0		
Umbilical hernia		4.8		1.7		8.9		
**Cardiac & genitourinary anomalies**								
Cardiac anomalies	113	19.5	69	21.7	44	15.9	0.492	^a^
ASD		10.6		13.0		6.8		
VSD		5.3		5.8		4.5		
Renal anomalies	95	12.6	53	11.3	42	14.3	0.666	^c^
Renal sonography, abnormal	43	25.6	19	21.1	24	29.2	0.105	^c^
Cryptorchidism	65	55.4	28	39.3	37	67.6	0.023	^a^
**Endocrinological abnormalities** ^e^								
Diabetes mellitus	71	7.0	43	7.0	28	7.1	1.000	^c^
Type 2 diabetes mellitus	4	75.0	2	100.0	2	50.0	1.000	^c^
Age (years) diagnosis (min–max)	2	18–46	1	18.0	1	46.0	0.317	^b^
Hypothyroidism	63	19.0	38	15.8	25	24.0	0.417	^a^
Age (years) diagnosis (median; min–max)	10	8; 1–40	4	4; 1.3–36.0	6	6; 1.0–40.0	0.394	^b^
Growth hormone deficiency	51	13.7	33	18.2	18	5.6	0.398	^c^
Growth hormone supplementation	50	12.0	31	16.1	19	5.3	0.387	^c^
**Behavioral characteristics**								
Behavioral abnormalities	71	83.1	28	85.7	43	81.4	0.945	^a^
Hyperkinetic		15.5		14.3		16.3		
Short attention		25.4		25.0		25.6		
Impulsiveness		14.1		14.3		14.0		
Obsessive		15.5		14.3		16.3		
Rigid		8.5		3.6		11.6		
Anger outbursts		16.9		10.7		20.9		
Aggressive		16.9		14.3		18.6		
Anxious		23.9		17.9		27.9		
Poor sociability		19.7		17.9		20.9		
Hyperactivity	63	42.9	25	60.0	38	31.6	0.026	^a^
High pain threshold^e^	47	40.4	28	28.6	19	57.9	0.044	^a^
**Psychiatric disorders**								
ADHD	48	33.3	16	50.0	32	25.0	0.083	^a^
Autistic traits	77	57.1	27	66.7	50	52.0	0.215	^a^
Malignancies	97	1.0	53	0.0	44	2.3	0.454	^c^

Only characteristics present in ≥5% of all patients or in either patient group, characteristics differing between groups, and distinctive features are shown.

*ADHD* attention deficit hyperactivity disorder, ARID1B-CSS patient group with a suspicion of Coffin–Siris syndrome before genetic testing, ARID1B-ID patient group with no suspicion of Coffin–Siris syndrome before genetic testing, *ASD* atrial septal defect, *CSS* Coffin–Siris syndrome, *EEG* electroencephalography, *ENT* ear nose throat, *OFC* occipitofrontal circumference, *SDS* standard deviation score, *VSD* ventricular septal defect.

^a^Chi-square.

^b^Mann–Whitney U.

^c^Fisher’s.

^d^The total number of a feature can differ from the sum of subcategories, because in some cases it was possible to answer with more than one option or to report the existence of a feature without specifying.

^e^Data regarding these features were collected through email after first analyses.

### Genotype

**Fig. 1:**
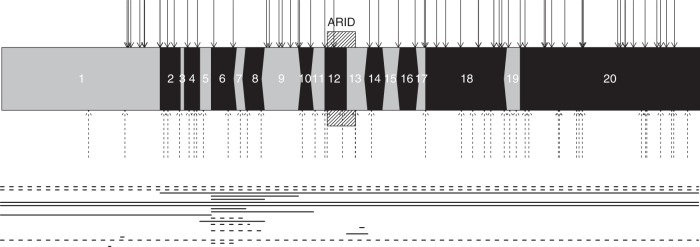
Overview of the location of pathogenic variants in *ARID1B*. Numbers represent exon numbers and a graphical representation of in-frame and out-frame exons. When an exon ends with a complete codon, a vertical line is displayed. If it has one additional base an arrow to the left is displayed, and two additional bases are indicated by an arrow to the right. In-frame exons thus have the same boundary on both sides of the exon. Small variants (defined as events ≤20 bases) are identified by the arrows above the exon structure; larger variants are shown as lines under the intron–exon structure. All large events were deletions. Only unique variants are shown. Uninterrupted lines represent variants in ARID1B-CSS patients; interrupted lines represent variants in ARID1B-ID patients.

Figure 1 and Supplementary Table S1 provide an overview of the submitted pathogenic variants. Sixty-two patients were previously reported in literature.^4–6,9,19,26^ Pathogenic variants were apparently de novo in all cases where parents could be tested (107/107). In two sisters the same pathogenic variant was found, while paternal DNA could not be obtained. Most pathogenic variants were frameshift or nonsense (*n* = 118), 18 were deletions involving multiple or all exons, and 7 involved canonical splice sites. One patient with a missense variant was initially submitted but later retracted when the variant turned out to be inherited from the unaffected father.

### Phenotype

Patients’ characteristics are summarized in Table 1 and Supplementary Table S2. Of the 143 individuals, 69 (48.3%) were female and the age at follow-up varied between 0 and 51 years with a median of 10 years. ARID1B-ID patients were less likely to be female than ARID1B-CSS patients (38 vs. 57%, *p* *=* 0.028). Two individuals have died, one of brain swelling potentially due to low-grade brain stem encephalitis that led to cardiorespiratory arrest at the age of 9 years, and one suddenly after the age of 24 years of unknown cause.

### Diagnosis before genetic testing

Seventy-nine ARID1B-CSS patients were included. Most (91.5%) of the 64 ARID1B-ID patients were retrospectively classified to fit the CSS spectrum by the referring clinician. An overview of the clinical diagnoses prior to identification of the pathogenic variant can be found in Supplementary Table S3. No statistically significant differences in phenotypic features were found between patients who were retrospectively classified to fit the CSS spectrum by their referring clinicians (*n* *=* 54) and those who were not (*n* *=* 5).

### Comparison of the ARID1B-ID and ARID1B-CSS groups

As expected, ARID1B-CSS patients more frequently displayed features associated with CSS than ARID1B-ID patients, including thick eyebrows, long eyelashes, thick alae nasi, long and/or broad philtrum, small nails and small or absent fifth distal phalanx, and hypertrichosis (*p* < 0.0001–0.001, Table 1).

ARID1B-ID patients appeared to have a higher prevalence of myopia (*p* *=* 0.020), cryptorchidism (*p* *=* 0.023), constipation (*p* *=* 0.002), sleep apnea (*p* = 0.026), hyperactivity (*p* = 0.026), and high pain threshold (*p* = 0.044), although these differences are not statistically significant under the Bonferroni adjusted significance level.

Regarding all other features, no significant differences were found between the ARID1B-CSS and ARID1B-ID groups (Table 1). Therefore, in the remainder of this section no distinction is made between both groups.

### Overall phenotype

#### Growth and development

**Fig. 2:**
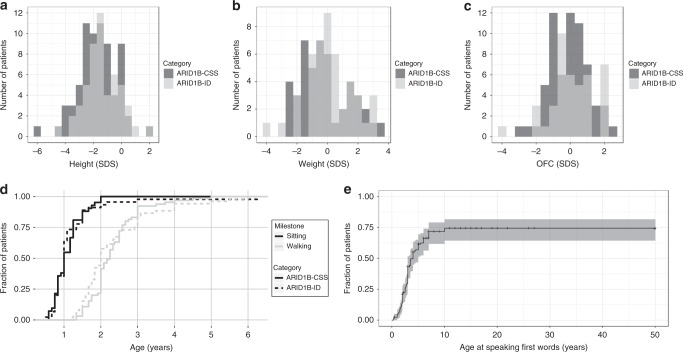
Biometry and developmental milestones. Histograms of the standard deviation score (SDS) of (**a**) height, *n* = 122; (**b**) weight, *n* = 92; and (**c**) occipitofrontal circumference (OFC), *n* = 105. (**d**) Cumulative distribution of the developmental milestones walking (*n* = 117) and sitting (*n* = 85) for ARID1B-CSS and ARID1B-ID. (**e**) Kaplan–Meier plot for the whole cohort of the age at which patients spoke their first words (*n* = 126). Confidence intervals of Kaplan–Meier plots are generated by R’s survfit function.

Histograms of the standard deviations of height, weight, and OFC are shown in Fig. 2a–c. A height below –2 SDS was observed in 30.3%. Confirming our previous data,^11^ the head circumference is normally distributed around 0 SDS and only 2.9% have an OFC below –2 SDS. Developmental milestones are shown in Fig. 2d, e. Speech, and gross and fine motor skills were delayed in almost all patients. Figure 2e shows the Kaplan–Meier plot of the age at first words, and this plot suggests that about 25% of patients do not develop speech.

Feeding difficulties were frequent (69.4%) and led to tube feeding in 16.9% of patients. Bone age was delayed in 47.5% and scoliosis occurred in 26.0% of patients. Frequent infections (57.3%) and gastrointestinal problems (48.6%, mostly constipation) were also reported.

#### Congenital anomalies

Cardiac anomalies (19.5%), cryptorchidism (55.4%), laryngomalacia (19.8%), and a nonfunctioning or absent tear duct (15.1%) were frequently reported. The cardiac anomalies consisted mostly of abnormalities/defects of the cardiac septa (*n* = 19) and/or mitral or aortic valves (*n* = 6). Renal anomalies were present in 12.6% of patients. The most frequent renal anomalies were hydronephrotic kidney (*n* = 3) and nephrolithiasis (*n* = 3).

#### Neurological features

**Fig. 3:**
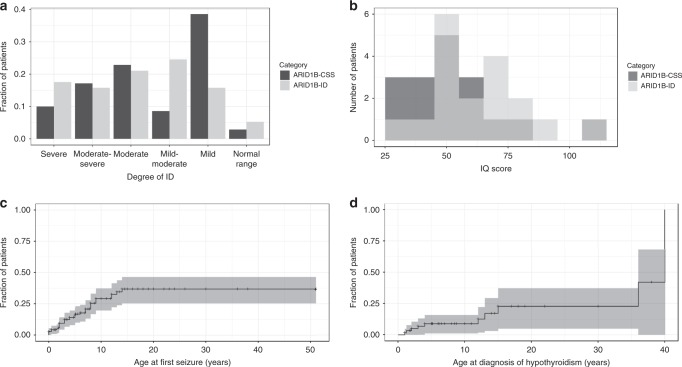
Degree of intellectual disability and survival analyses of seizures and hypothyroidism. (**a**) Intellectual disability (ID) category as assessed by the treating physician, *n* = 127; and (**b**) IQ scores (determined at different ages using different scales), *n* = 35 for ARID1B-CSS and ARID1B-ID patients. (**c**) Kaplan–Meier plot for age of onset of seizures, *n* = 37 and (**d**) Kaplan–Meier plot for the age at which hypothyroidism was diagnosed, *n* = 10. Confidence intervals of Kaplan–Meier plots are generated by R’s survfit function.

Almost all patients exhibited a variable degree of ID. Figure 3a, b show the distribution of reported ID severity and IQ scores (*n* *=* 35). Remarkably, several patients did have an IQ in the normal range.

Hypotonia occurred in 81.0% of patients and 27.5% suffered from seizures, while an additional 5.6% had an abnormal electroencephalogram (EEG). The age of onset of the seizures varied from 0 to 14 years, with a median of 4 years (Fig. 3c). The Kaplan–Meier plot suggests that about 40% of patients are expected to experience one or more epileptic seizure during their life. All patients responded well to seizure medication (*n* *=* 18), while 4 did not receive medication. Complete or partial ACC (42.6%) and delayed myelination (17.0%) were the most prevalent cerebral anomalies.

#### Visual and hearing impairment

Vision problems (48.6%) were twice as frequent as hearing problems (22.1%). Myopia was the most reported vision problem (27.5%); severity ranged from –24 to –1.0 (median –6.35). Hearing loss was congenital in most patients (63.6%) and when present usually bilateral (51.9%); four patients needed hearing aids.

#### Endocrinological features

Hypothyroidism was documented in 19.0% and one patient had hypothyroidism for 2 years, which spontaneously resolved. The Kaplan–Meier plot of age at which hypothyroidism developed (Fig. 3d) suggests that the prevalence may be 25%, but because the numbers are small there is a large confidence interval. Of 71 individuals, 5 (7.0%) were diagnosed with diabetes, 1 with hyperinsulinism, and 7/51 (13.7%) had a growth hormone (GH) deficiency, of which 6 received GH supplementation. We had no information on whether GH deficiency was partial or complete.

### Effect of location of pathogenic variant

The location of the pathogenic variant did not appear to correlate with the severity of the phenotype (Supplementary Figure S2). Because it has been reported that patients with pathogenic variants in in-frame exons sometimes have less severe phenotypes because of naturally occurring exon skipping,^27^ we compared the phenotypes of patients with pathogenic variants in in-frame exons versus those with pathogenic variants in out-frame exons. We classified patients with pathogenic variants in the first and last exon and whole-gene deletions as out-frame, because such pathogenic variants cannot be rescued by exon skipping. Most pathogenic variants were classified as out-frame (84.6%), which was expected because 86.1% of the coding region consists of out-frame exons. We did not find any differences in the phenotype between these two groups.

## DISCUSSION

### Genotype

Information on 143 patients with pathogenic variants in *ARID1B* was collected. In accordance with previous studies, all pathogenic variants were truncating (nonsense, frameshift, splice-site, and deletions of various numbers of exons including whole-gene deletions). In addition, four translocations^14,15,28,29^ and three duplications^2,16,21^ affecting *ARID1B* have been reported in literature. While previous reviews^30,31^ have suggested that a pathogenic missense variant was reported in Tsurusaki et al.,^9^ it is worth noting that a nonsense variant in *ARID1B* was also found in the same patient. Although parental DNA was not available for this patient, it seems most likely that the missense variant was harmless, while the nonsense variant was causal. Yu et al.^16^ described two pathogenic de novo missense variants in patients with short stature, but without ID or speech delay. Mignot et al.^32^ described a patient with mild ID and ACC who inherited a pathogenic missense variant from a mildly affected mother. Because the pathogenic variant (c.6092 T>C; p.Ile2031Thr) arose de novo in the mother, it is highly likely to be pathogenic. Thus, missense variants appear to be a much less common cause of ID than truncating variants. Missense variants identified in the absence of parental DNA should be interpreted with caution and are much more likely to be harmless. Care should also be taken with the interpretation of de novo missense variants in patients with ID.

As suggested by Johnston et al.,^33^ it is important to take note if pathogenic variants in the same exon have been previously described when interpreting variants, because some annotated exons might in fact not be relevant for disease-associated transcripts. It is therefore noteworthy that we have not identified any pathogenic variants that only affect exon 3 (Fig. 1). Exon 3 is a small in-frame exon and we previously indicated^11^ that truncating variants in this exon are most likely benign. Indeed, Johnston et al.^33^ report an inherited truncating variant in exon 3 in a patient without an ARID1B-related phenotype. Taking all evidence together, it is most likely that pathogenic variants in exon 3 are nonpathogenic, and that transcript NM_017519.2, which lacks exon 3 of NM_020732.3, is in fact the more relevant transcript. Another remarkable aspect that is apparent from Fig. 1 is that no pathogenic variants have been reported in the first 849 bases of *ARID1B*. Although some frameshift variants are reported in gnomAD, these are dubious calls because the variants are in a low-complexity region. The lack of pathogenic variants may be by chance or due to sequencing difficulties in this GC-rich area, but it is also possible that there is an alternative start site that renders truncating variants in the first part of exon 1 neutral. Therefore, we advise caution when interpreting such variants.

Although a previous analysis seemed to suggest that pathogenic variants in the last exon might result in a more severe phenotype,^11^ repeating this analysis with the current data did not confirm this suspicion (Supplementary Figure S2).

### Penetrance

Penetrance of rare variants cannot be directly estimated, but the inheritance status and prevalence of apparently pathogenic variants in population databases can be used as proxy.^34^ Pathogenic variants were de novo in all cases where inheritance could be established (*n* = 107). We did observe two sisters with the same pathogenic variant, and although this could be due to paternal inheritance, the most likely explanation is gonadal mosaicism, as previously described for *ARID1B*.^17^

The gnomAD browser may be viewed as a large population data set, consisting of exomes and genomes of unrelated, unaffected individuals sequenced as part of disease-specific and population genetic studies. In transcript NM_020732.2 (ENST00000346085), fewer missense variants in *ARID1B* are reported than would be expected if these were to occur randomly (ExAC accessed 10 April 2018; 744.4 expected, 555 observed, *z*-score of 3.39). Ten loss-of-function variants are reported; half of these might not be pathogenic because they are either in the start of exon 1, or in exon 3 (Supplementary Table S5). The other five variants, three splice-site variants and two frameshift variants, are potentially pathogenic. This might suggest that the penetrance of pathogenic variants in *ARID1B* is not complete, and that these individuals had an average IQ as some of the individuals in our study. Therefore, while the penetrance of pathogenic variants in *ARID1B* appears to be very high, we still recommend parental testing, especially when future pregnancies are considered.

### Phenotype

The main differences between ARID1B-CSS and ARID1B-ID seem to be related to dysmorphic features. Therefore, we conclude from this data set that the ARID1B-related disorders represent a spectrum. Not every feature is present in all patients, and depending on the combination of features present, and the experience of the clinician concerned, a patient might receive a clinical diagnosis of CSS. Although the patients in our previous *ARID1B-CSS* cohort^11^ had an equal sex ratio, our current cohort finds significantly more females than males in the *ARID1B-CSS* group compared with the ARID1B-ID group. This may be because some features, most notably hypertrichosis, are easier to recognize as abnormal in females than in males.

### Endocrinological features

Hypothyroidism was reported in 12/63 patients, and most were diagnosed before the age of 15 years (Fig. 3d). Diabetes was diagnosed in 5/71 patients; 4 of these patients were reported to have type 2 diabetes and had a relatively high weight, while one was diagnosed with type 1 diabetes, and 1 patient was diagnosed with hyperinsulinism. This patient with hyperinsulinism has been described previously,^19^ and one additional patient has been reported to have hyperinsulinism.^10^ Of our patients, 7/51 were diagnosed with GH deficiency, and 6 of those received GH supplementation. Likewise, several patients with a growth delay due to GH deficiency were described^16^ and a similar phenomenon was replicated in an Arid1b heterozygous mouse model.^35^ Considering 30% of patients had short stature (<–2 SDS),^36^ GH deficiency could be an underrecognized feature of ARID1B patients.

### Cancer

Somatic variants in *ARID1B* have been associated with several types of cancer.^37^ However, only one case is known of a patient with a pathogenic germline variant in *ARID1B* and (thyroid) cancer.^38^ Similarly, in our cohort only one patient had malignancies. This boy had a Sertoli–Leydig cell tumor at the age of 3 and a temporal glioneuronal tumor at 12 years of age. No genetic testing was performed in this patient to detect the presence of specific tumor syndromes. Based on our patient cohort it seems unlikely that pathogenic germline variants in *ARID1B* confer an increased cancer risk, but longer follow-up of our patients is needed to make a definitive statement.

### Phenotype delineation methods and biases

The increased awareness of ascertainment bias and the increasing number of newly discovered disease genes have resulted in new methods for data collection. Paper questionnaires and Excel spreadsheets are rapidly being replaced by Internet forms and HPO-based methods along with more formal registry software interfaces such as PhenoTips.^39^ For the current study we have chosen to use an online questionnaire based on our previous data, with mostly fixed answers. More general genetic databases (such as DECIPHER^40^) accommodate inclusion of phenotypic data by using HPO terms. Whereas HPO-based methods allow for more straightforward identification of new, unexpected findings, our approach with specific questions allows calculation of true frequencies of features, because we can discriminate between absence of a feature and missing data. To investigate whether this influences results, we compared our data regarding the phenotype of ARID1B patients with the features reported for ARID1B patients in the DECIPHER database. It should be noted that there are some patients who are both in our cohort and the DECIPHER database. On 2 October 2017 DECIPHER contained 54 open-access ARID1B patients with 247 phenotypic features. Except for laryngomalacia and excessive salivation and/or drooling, all characteristics enriched in ARID1B patients in DECIPHER had been included in our questionnaire. The DECIPHER frequency of all reported features was lower than ours, a difference that was statistically significant for most (Supplementary Table S6).

In addition to other phenotypic features, growth and development are graphically represented on the DECIPHER website. We replicated some of these graphs and found only minor differences, which may be explained by the increased volume of data available to us (Supplementary Table S7 and Supplementary Figure S3).

Based on these results, we conclude that the DECIPHER database is a valuable tool to assess the potential features that ought to be included in gene-specific phenotype questionnaires, but the reported percentages are potential underestimations, likely due to phenotype underreporting bias owing to their data collection method. This bias could be mitigated by developing an adaptive questionnaire, so that submitters are requested to include or actively exclude features that have been mentioned several times in patients with the same underlying genetic cause.

Another bias that is present in most cohorts is an age-related bias. Most of our population is young, and this precludes us from detecting features that typically present at later age. This bias can partially be resolved by performing survival analysis, as we have done for speech, seizures, and hypothyroidism. In all cases there was a clear difference in our global frequency estimate, and the estimate provided by the Kaplan–Meier plot. We therefore recommend that authors of clinical cohorts include the age of occurrence of age-dependent features and perform survival analysis.

### Study limitations

Our study has several limitations. Data entry was performed by many different clinicians, which could have led to different interpretation of subjective questions, such as whether the patient fits the CSS spectrum. During this study, it became evident from literature and the DECIPHER database that previously unknown features, such as laryngomalacia, were part of the ARID1B spectrum and should have been added to our questionnaire. The effect of these incompletions was mitigated in part by the presence of an open field and our request to the contributing clinicians to send us an update on their patient’s characteristics for frequently reported additional features.

Although we have attempted to account for different sources of bias, we cannot exclude that we currently overestimate the presence of some features, because our calculation is based on those patients having the feature, divided by those who are reported not to have the feature. It is possible that in some cases the absence of a feature is not consciously recognized or recorded in the clinical charts, which may result in scoring “unknown” rather than “absent” for a given feature. This bias can only be mitigated by thoroughly phenotyping every patient by a limited number of physicians, and is something we are planning to do with our national CSS cohort in the near future.

### Conclusion

We conclude that the ARID1B-related disorders encompass a spectrum of features. The typical ARID1B patient has ID, feeding difficulties, laryngomalacia, speech delay, motor delay, hypertrichosis, and cryptorchidism. Our data suggest that endocrinological abnormalities, in particular hypothyroidism, may also be part of the ARID1B spectrum, but further research is needed to confirm this finding. There are few differences between ARID1B-CSS and ARID1B-ID, and we recommend that patients should be managed similarly. Based on the clinical data presented here we update our previous recommendations^11^ for the management of ARID1B patients:

#### At the time of diagnosis

- Rule out congenital anomalies by performing renal and cardiac ultrasounds. Thoroughly check for cryptorchidism and laryngomalacia as indicated.
- Refer to clinical geneticist for counseling.

#### At the time of diagnosis and at follow-up

- Evaluate growth; consider referral to endocrinologist when significantly delayed.
- Evaluate hearing and vision regularly.
- Consider an EEG if there is a suspicion of seizures.
- Evaluate feeding problems. Offer dietary advice and feeding management. Consider evaluation by a gastroenterologist and/or a swallowing study.
- Treat constipation adequately.
- If indicated, early intervention using speech and/or physical therapy.
- Yearly evaluation of development by a specialized pediatrician and implementation of a specialized education plan for school or daycare as indicated.
- Periodic evaluations for scoliosis.
- On indication, determine thyroid status and glucose concentrations.

We also recommend periodic evaluation by a team of professionals specializing in ARID1B-related disorders (e.g., pediatrician, pediatric physiatrist, physical therapist, speech therapist, behavioral specialist, pediatric neurologist, clinical geneticist), which may take place at a distance.

## Electronic supplementary material

Supplementary Information
