# Correction: The ARID1B spectrum in 143 patients: from nonsyndromic intellectual disability to Coffin–Siris syndrome

**DOI:** 10.1038/s41436-018-0368-y

**Published:** 2019-01-29

**Authors:** Pleuntje J. van der Sluijs, Sandra Jansen, Samantha A. Vergano, Miho Adachi-Fukuda, Yasemin Alanay, Adila AlKindy, Anwar Baban, Allan Bayat, Stefanie Beck-Wödl, Katherine Berry, Emilia K. Bijlsma, Levinus A. Bok, Alwin F. J. Brouwer, Ineke van der Burgt, Philippe M. Campeau, Natalie Canham, Krystyna Chrzanowska, Yoyo W. Y. Chu, Brain H. Y. Chung, Karin Dahan, Marjan De Rademaeker, Anne Destree, Tracy Dudding-Byth, Rachel Earl, Nursel Elcioglu, Ellen R. Elias, Christina Fagerberg, Alice Gardham, Blanca Gener, Erica H. Gerkes, Ute Grasshoff, Arie van Haeringen, Karin R. Heitink, Johanna C. Herkert, Nicolette S. den Hollander, Denise Horn, David Hunt, Sarina G. Kant, Mitsuhiro Kato, Hülya Kayserili, Rogier Kersseboom, Esra Kilic, Malgorzata Krajewska-Walasek, Kylin Lammers, Lone W. Laulund, Damien Lederer, Melissa Lees, Vanesa López-González, Saskia Maas, Grazia M. S. Mancini, Carlo Marcelis, Francisco Martinez, Isabelle Maystadt, Marianne McGuire, Shane McKee, Sarju Mehta, Kay Metcalfe, Jeff Milunsky, Seiji Mizuno, John B. Moeschler, Christian Netzer, Charlotte W. Ockeloen, Barbara Oehl-Jaschkowitz, Nobuhiko Okamoto, Sharon N. M. Olminkhof, Carmen Orellana, Laurent Pasquier, Caroline Pottinger, Vera Riehmer, Stephen P. Robertson, Maian Roifman, Caroline Rooryck, Fabienne G. Ropers, Monica Rosello, Claudia A. L. Ruivenkamp, Mahmut S. Sagiroglu, Suzanne C. E. H. Sallevelt, Amparo Sanchis Calvo, Pelin O. Simsek-Kiper, Gabriela Soares, Lucia Solaeche, Fatma Mujgan Sonmez, Miranda Splitt, Duco Steenbeek, Alexander P. A. Stegmann, Constance T. R. M. Stumpel, Saori Tanabe, Eyyup Uctepe, G. Eda Utine, Hermine E. Veenstra-Knol, Sunita Venkateswaran, Catheline Vilain, Catherine Vincent-Delorme, Anneke T. Vulto-van Silfhout, Patricia Wheeler, Golder N. Wilson, Louise C. Wilson, Bernd Wollnik, Tomoki Kosho, Dagmar Wieczorek, Evan Eichler, Rolph Pfundt, Bert B. A. de Vries, Jill Clayton-Smith, Gijs W. E. Santen

**Affiliations:** 1 Department of Clinical Genetics, Leiden University Medical Center, Leiden, The Netherlands; 2 Department of Human Genetics, Donders Institute for Brain, Cognition and Behaviour, Radboud University Medical Center, Nijmegen, The Netherlands; 3 Division of Medical Genetics and Metabolism, Children’s Hospital of the King’s Daughters, Norfolk, VA USA; 4 Department of Pediatrics, St. Marianna University School of Medicine, Kanagawa, Japan; 5 School of Medicine, Department of Pediatrics, Pediatric Genetics Unit, Acibadem University, Istanbul, Turkey; 6 Department of Genetics, Sultan Qaboos University Hospital, Muscat, Oman; 7 Pediatric Cardiology and Cardiac Surgery Department, Bambino Gesù Children Hospital and Research Institute, IRCCS, Rome, Italy; 8 Copenhagen University Hospital Hvidovre, Copenhagen, Denmark; 9 Department of Molecular Genetics and Applied Genomics, University Hospital Tübingen, Tübingen, Germany; 10 Department of Medical Genetics, Shodair Hospital, Helena, MT USA; 11 Department of Pediatrics, Màxima Medical Centre, Veldhoven, The Netherlands; 12 Department of Paediatrics, Nij Smellinghe Hospital, Drachten, The Netherlands; 13 Department of Human Genetics, Radboud University Medical Center, Nijmegen, The Netherlands; 14 Department of Pediatrics, CHU Sainte-Justine and University of Montreal, Montreal, QC Canada; 15 North West Thames Regional Genetics Service, Northwick Park Hospital, Harrow, United Kingdom; 16 Cheshire and Merseyside Regional Genetics Service, Liverpool Women’s Hospital, Crown Street, Liverpool, United Kingdom; 17 Department of Medical Genetics, The Children’s Memorial Health Institute, Warsaw, Poland; 18 Department of Paediatrics and Adolescent Medicine, Li Ka Shing Faculty of Medicine, The University of Hong Kong, Hong Kong, SAR China; 19 Center for Human Genetics, Institute of Pathology and Genetics, Gosselies, Belgium; 20 Center for Medical Genetics, Vrije Universiteit Brussels, Brussels, Belgium; 21 Hunter Genetics and University of Newcastle, GrowUpWell Priority Research Centre, Newcastle, Australia; 22 Department of Psychiatry and Behavioral Sciences, University of Washington, Seattle, WA USA; 23 Department of Pediatric Genetics, Marmara University Pendik Hospital, Istanbul, Turkey; 24 Department of Pediatrics and Genetics, University of Colorado Denver School of Medicine, Aurora, CO USA; 25 Department of Clinical Genetics, Odense University Hospital, Odense, Denmark; 26 Department of Genetics, Cruces University Hospital, Biocruces Health Research Institute, Vizcayam, Spain; 27 University of Groningen, University Medical Center Groningen, Department of Genetics, Groningen, The Netherlands; 28 Department of Rehabilitation Medicine, Leiden University Medical Center, Leiden, The Netherlands; 29 Institute for Medical Genetics and Human Genetics, Charité Universitätsmedizin, Berlin, Germany; 30 Wessex Clinical Genetics Service, Princess Anne Hospital, Southampton, United Kingdom; 31 Department of Pediatrics, Showa University School of Medicine, Tokyo, Japan; 32 Medical Genetics Department, Koç University School of Medicine (KUSoM), İstanbul, Turkey; 33 Department of Clinical Genetics, Sophia Children’s Hospital, Erasmus MC, Rotterdam, The Netherlands; 34 Department of Pediatric Genetics, Hematology Oncology Research & Training Children’s Hospital, Ankara, Turkey; 35 Department of Medical Genetics, Dayton Children’s Hospital, Dayton, OH USA; 36 Department of Paediatrics, Odense University Hospital, Odense, Denmark; 37 Department of Clinical Genetics, Great Ormond Street Hospital NHS Foundation Trust, London, United Kingdom; 38 Sección de Genética Médica, Servicio de Pediatria, Hospital Clinico Universitario Virgen de la Arrixaca, IMIB-Arrixaca, CIBERERISCIII, Murcia, Spain; 39 Department of Clinical Genetics, Academic Medical Center, University of Amsterdam, Amsterdam, The Netherlands; 40 Unidad de Genética, Hospital Universitario y Politécnico La Fe, Valencia, Spain; 41 Department of Molecular and Human Genetics, Baylor College of Medicine, One Baylor Plaza, Houston, TX USA; 42 Northern Ireland Regional Genetics Centre, Belfast City Hospital, Belfast, Ireland; 43 East Anglian Regional Genetics Service, Cambridge University Hospitals NHS Foundation Trust, Addenbrooke’s Hospital, Cambridge, United Kingdom; 44 Manchester Centre for Genomic Medicine, Division of Evolution and Genomic Sciences, St Mary’s Hospital, Manchester University Hospitals NHS Foundation Trust Manchester Academic Health Sciences Centre, Manchester, United Kingdom; 45 Center for Human Genetics Inc, Cambridge, MA USA; 46 Department of Pediatrics, Central Hospital, Aichi Human Service Center, Kasugai, Aichi Japan; 47 Department of Pediatrics, Geisel School of Medicine, Dartmouth College, Hanover, NH USA; 48 Institute of Human Genetics, University Hospital of Cologne, Cologne, Germany; 49 Gemeinschaftspraxis für Humangenetik Homburg/Saar, Homburg, Germany; 50 Department of Medical Genetics, Osaka Women’s and Children’s Hospital, Osaka, Japan; 51 Willem Alexander Children’s Hospital, Leiden University Medical Center, Leiden, The Netherlands; 52 CRMR Déficiences intellectuelles, Service de Génétique Médicale, CLAD Ouest CHU Hôpital Sud, Rennes, France; 53 All Wales Medical Genetics Service, Glan Clwyd Hospital, Rhyl, United Kingdom; 54 Dunedin School of Medicine, University of Otago, Dunedin, New Zealand; 55 Division of Clinical and Metabolic Genetics, Department of Paediatrics, The Hospital for Sick Children, University of Toronto, Toronto, ON Canada; 56 The Prenatal Diagnosis and Medical Genetics Program, Department of Obstetrics and Gynecology, Mount Sinai Hospital, Toronto, ON Canada; 57 Department of Medical Genetics, CHU Bordeaux, Bordeaux, France; 58 Department of Pediatrics, Leiden University Medical Center, Leiden, The Netherlands; 59 Genpute Computation Technologies Company, Istanbul, Turkey; 60 Department of Clinical Genetics and GROW-School for Oncology and Developmental Biology, Maastricht University Medical Center, Maastricht, The Netherlands; 61 Servicio de Pediatría, Hospital Universitario Doctor Peset, Valencia, Spain; 62 Department of Pediatric Genetics, Ihsan Dogramaci Children’s Hospital, Hacettepe University School of Medicine, Ankara, Turkey; 63 Jacinto de Magalhães Medical Genetics Center, Centro Hospitalar do Porto, Porto, Portugal; 64 Departamento de neurometabólicas, Hospital Universitario Son Espases, Palma de Mallorca, Spain; 65 Karadeniz Technical University, Faculty of Medicine, Dept of Child Neurology, Retired Professor, Trabzon, Turkey; 66 Northern Genetics Service, Institute of Genetics Medicine, Newcastle upon Tyne, United Kingdom; 67 Division of Pediatrics, Yamagata Prefectural and Sakata Munici pal Hospital Organization Nihon-Kai General Hospital, Sakata, Japan; 68 Enva Engineering, Ankara, Turkey; 69 Division of Neurology, Department of Pediatrics, Children’s Hospital of Eastern Ontario, University of Ottawa, Ottawa, ON Canada; 70 Department of Genetics, Hôpital Universitaire des Enfants Reine Fabiola, ULB Center of Medical Genetics, Université Libre de Bruxelles, Brussels, Belgium; 71 Department of Genetics, Hôpital Erasme. ULB Center of Medical Genetics, Université Libre de Bruxelles, Brussels, Belgium; 72 Service de génétique clinique Guy Fontaine, CHRU de Lille–Hôpital Jeanne de Flandre, Lille, France; 73 Division of Genetics, Arnold Palmer Hospital, Orlando, FL USA; 74 KinderGenome Genetics, Medical City Hospital Dallas, Dallas, TX USA; 75 Institute of Human Genetics, University Medical Center Göttingen, Göttingen, Germany; 76 Center for Medical Genetics, Shinshu University Hospital, Matsumoto, Japan; 77 Institute of Human Genetics, Medical Faculty, Heinrich-Heine-University, Düsseldorf, Germany; 78 Department of Genome Sciences, University of Washington School of Medicine, Seattle, WA USA

Correction to: *Genetics in Medicine* 10.1038/s41436-018-0330-z online publication 8 November 2018

The original version of this Article contained an error in the spelling of the author Pleuntje J. van der Sluijs, which was incorrectly given as Eline (P. J.) van der Sluijs. This has now been corrected in both the PDF and HTML versions of the Article.

