# Supplementary material for: The ARID1B spectrum in 143 patients: from nonsyndromic intellectual disability to Coffin–Siris syndrome
Source: Genet Med. 2018 Nov 8;21(6):1295–307. doi: 10.1038/s41436-018-0330-z (PMC6752273; doi:10.1038/s41436-018-0330-z)
Supplement: Supplementary file 1 — Supplementary Information [file 41436_2018_330_MOESM1_ESM.pdf]

SUPPLEMENTARY MATERIAL

The ARID1B spectrum in 143 patients: From non-syndromic intellectual disability to Coffin-Siris syndrome

Eline (P.J.) van der Sluijs, Sandra Jansen, Samantha A. Vergano, Miho Adachi-Fukuda, Yasemin Alanay, Adila AlKindy, Anwar Baban, Allan Bayat, Stefanie Beck-Wödl, Katherine Berry, Emilia K. Bijlsma, Levinus A. Bok, Alwin F.J. Brouwer, Ineke van der Burgt, Philippe M. Campeau, Natalie Canham', Krystyna Chrzanowska, Yoyo W.Y. Chu, Brain H.Y. Chung, Karin Dahan, Marjan De Rademaeker, Anne Destree, Tracy Dudding-Byth, Rachel Earl, Nursel Elcioglu, Ellen R. Elias, Christina Fagerberg, Alice Gardham, Blanca Gener, Erica H. Gerkes, Ute Grasshoff, Arie van Haeringen, Karin R. Heitink, Johanna C. Herkert, Nicolette S. den Hollander, Denise Horn, David Hunt, Sarina G. Kant, Mitsuhiro Kato, Hülya Kayserili, Rogier Kersseboom, Esra Kilic, Malgorzata Krajewska-Walasek, Kylin Lammers, Lone W. Laulund, Damien Lederer, Melissa Lees, Vanesa López-González, Saskia Maas, Grazia M.S. Mancini, Carlo Marcelis, Francisco Martinez, Isabelle Maystadt, Marianne McGuire, Shane McKnee, Sarju Mehta, Kay Metcalfe, Jeff Milunsky, Seiji Mizuno, John B. Moeschler, Christian Netzer, Charlotte W. Ockeloen, Barbara Oehl-Jaschkowitz, Nobuhiko Okamoto, Sharon N.M. Olminkhof, Carmen Orellana, Laurent Pasquier, Caroline Pottinger, Vera Riehmer, Stephen P. Robertson, Maian Roifman', Caroline Rooryck, Fabienne G. Ropers, Monica Rosello, Claudia A.L. Ruivenkamp, Mahmut S. Sagiroglu, Suzanne C.E.H. Sallevelt, Amparo Sanchis Calvo, Pelin O. Simsek-Kiper, Gabriela Soares, Lucia Solaeche, Fatma Mujgan Sonmez, Miranda Splitt, Duco Steenbeek, Alexander P.A. Stegmann, Constance T.R.M. Stumpel, Saori Tanabe, Eyyup Uctepe, G. Eda Utine, Hermine E. Veenstra-Knol, Sunita Venkateswaran, Catheline Vilain', Catherine Vincent-Delorme, Anneke T. Vulto-van Silfhout, Patricia Wheeler, Golder N. Wilson, Louise C. Wilson, Bernd Wollnik, Tomoki Kosho, Dagmar Wieczorek, Evan Eichler, Rolph Pfundt, Bert B.A. de Vries, Jill Clayton-Smith, and Gijs W.E. Santen

Contents

- Table S1 p. 3
- Table S2 p. 7
- Table S3 p.10
- Table S4 p.11
- Table S5 p.12
- Table S6 p.13
- Figure S1 p.14
- Figure S2 p.15
- Figure S3 p.16

Table S1 Identified pathogenic variants in ARID1B

#	Exon	cdnachange (NM_020732.3(ARID1B):)	Protein change	Inheritance	Type of mutation	gdna (Chr6(GRCh37):)	Group	Reference
75	1-20	c.-2162464_*1205821del	p.0	Unknown	Deletion	g.154936600_158734846del	ARID1B-CSS	
137	1-20	c.-441222_*437696del	p.0	Unknown	Deletion	g.156657842_157966721del	ARID1B-ID	
20	1-20	c.-392315_*2903306del	p.0	De novo	Deletion	g.156706749_160432331del	ARID1B-CSS	(Tsurusaki et al., 2012)
65	1-20	c.-138625_*1360628del	p.0	De novo	Deletion	g.156960439_158889653del	ARID1B-CSS	(Wieczorek et al., 2013)
86	1-5	c.-39637_2037+61693del	p.0	Unknown	Deletion	g.157059427_157318403del	ARID1B-ID	
9	1-20	c.(?_-1)_(*1_?)del	p.0	Unknown	Deletion	_	ARID1B-CSS	(Santen et al., 2013)
104	1-20	c.(?_-1)_(*1_?)del	p.0	Unknown	Deletion	_	ARID1B-CSS	
140	1	c.850C>T	p.Gln284*	De novo	Nonsense	g.157099913C>T	ARID1B-ID	
146	1	c.1044_1065del	p.Gly351Metfs*11	Unknown	Frameshift	g.157100107_157100128del	ARID1B-ID	
135	1	c.1160_1200del	p.Ala387Glyfs*134	De novo	Frameshift	g.157100223_157100263del	ARID1B-ID	
133	1	c.1202del	p.Gly401Alafs*29	De novo	Frameshift	g.157100265del	ARID1B-ID	
7	1	c.1222dup	p.Gln408Profs*127	De novo	Frameshift	g.157100285dup	ARID1B-CSS	(Santen et al., 2013)
39	1	c.1235dup	p.Ser413Valfs*122	De novo	Frameshift	g.157100298dup	ARID1B-CSS	(Santen et al., 2013)
6	1	c.1259dup	p.Asn420Lysfs*115	De novo	Frameshift	g.157100322dup	ARID1B-CSS	(Santen et al., 2013)
5	1	c.1346del	p.Pro449Argfs*53	De novo	Frameshift	g.157100409del	ARID1B-CSS	(Santen et al., 2013)
68	1	c.1389_1398del	p.Ala464Serfs*35	De novo	Frameshift	g.157100452_157100461del	ARID1B-CSS	(Tsurusaki et al., 2014)
82	1	c.1389_1398del	p.Ala464Serfs*35	De novo	Frameshift	g.157100452_157100461del	ARID1B-ID	
124	1	c.1389_1398del	p.Ala464Serfs*35	De novo	Frameshift	g.157100452_157100461del	ARID1B-CSS	
21	1	c.1392_1402del	p.Gln467Argfs*64	De novo	Frameshift	g.157100455_157100465del	ARID1B-CSS	(Tsurusaki et al., 2014)
50	1	c.1540dupC	p.Gln514Profs*21	De novo	Frameshift	g.157100603dup	ARID1B-CSS	(Wieczorek et al., 2013)
4	1	c.1542+1G>A	r.spl	De novo	Splice site	g.157100606G>A	ARID1B-CSS	(Santen et al., 2013)
23	2-20	c.1543-5717_*499944del	p.0	De novo	Deletion	g.157144644_158028969del	ARID1B-ID	
127	2	c.1579C>T	p.Gln527*	De novo	Nonsense	g.157150397C>T	ARID1B-ID	
76	2	c.1612C>T	p.Gln538*	De novo	Nonsense	g.157150430C>T	ARID1B-CSS	
97	2	c.1618C>T	p.Gln540*	De novo	Nonsense	g.157150436C>T	ARID1B-ID	
14	2	c.1678_1688del	p.Ile560Glyfs*89	De novo	Frameshift	g.157150496_157150506del	ARID1B-CSS	(Tsurusaki et al., 2012)
77	2	c.1729C>T	p.Gln577*	De novo	Nonsense	g.157150547C>T	ARID1B-ID	
51	4	c.1808dupG	p.Ser603Argfs*50	De novo	Frameshift	g.157222541dup	ARID1B-CSS	(Wieczorek et al., 2013)
142	4	c.1822C>T	p.Gln608*	De novo	Nonsense	g.157222555C>T	ARID1B-ID	
119	4	c.1871del	p.Pro624Hisfs*44	De novo	Frameshift	g.157222604del	ARID1B-ID	(Wieczorek et al., 2013)
36	4	c.1897C>T	p.Gln633*	De novo	Nonsense	g.157222630C>T	ARID1B-ID	
15	4	c.1903C>T	p.Gln635*	De novo	Nonsense	g.157222636C>T	ARID1B-CSS	(Tsurusaki et al., 2012)
95	5-8	c.1927-13575_2552-1769del	p.0	De novo	Deletion	g.157243025_157467989del	ARID1B-CSS	
143	5	c.1960C>T	p.Gln654*	Unknown	Nonsense	g.157256633C>T	ARID1B-ID	
31	6-9	c.(2037+1_2038-1)_ (2879+1_2880-1)del	p.0	De novo	Deletion	_	ARID1B-CSS	(Santen et al., 2013)
101	6,7	c.2038-144324_2407del	p.?	De novo	Deletion	g.157261472_157454197del	ARID1B-ID	
85	6-10	c.2038-144294_3025+3178del	p.0	De novo	Deletion	g.157261502_157491497del	ARID1B-ID	

131	6-8	c.2038-125635_2551+6049del	p.Asp680Valfs*63	Unknown	Deletion	g.157280161_157460390del	ARID1B-ID	
144	6	c.2038-100946_2281+309del	p.0	De novo	Deletion	g.157304850_157406347del	ARID1B-ID	
80	6,7	c.2038-52488_2371+613del	p.Asp680Valfs*54	Unknown	Deletion	g.157353308_157432308del	ARID1B-ID	
89	6,7	c.2038-52488_2371+613del	p.Asp680Valfs*54	Unknown	Deletion	g.157353308_157432308del	ARID1B-ID	
64	6-8	c.2038-3756_2551+6201del	p.Asp680Valfs*63	De novo	Deletion	g.157402040_157460542del	ARID1B-CSS	(Wieczorek et al., 2013)
71	6	c.2062del	p.Leu688Serfs*9	De novo	Frameshift	g.157405820del	ARID1B-CSS	(Tsurusaki et al., 2014)
98	6	c.2200_2201delinsT	p.Gly734Serfs*11	De novo	Frameshift	g.157405958_157405959delinsT	ARID1B-ID	
52	6	c.2248C>T	p.Arg750*	De novo	Nonsense	g.157406006C>T	ARID1B-CSS	(Wieczorek et al., 2013)
53	6	c.2248C>T	p.Arg750*	De novo	Nonsense	g.157406006C>T	ARID1B-CSS	(Wieczorek et al., 2013)
129	6	c.2248C>T	p.Arg750*	Unknown	Nonsense	g.157406006C>T	ARID1B-CSS	
84	7	c.2318C>G	p.Ser773*	De novo	Nonsense	g.157431642C>G	ARID1B-ID	
139	8	c.2385dup	p.Thr796Hisfs*35	De novo	Frameshift	g.157454175dup	ARID1B-ID	
88	8	c.2519dup	p.Tyr840*	De novo	Frameshift	g.157454309dup	ARID1B-ID	
141	9	c.2572_2573delinsCTGG	p.Ala858Leufs*57	De novo	Frameshift	g.157469778_ 157469779delinsCTGG	ARID1B-CSS	
43	9	c.2598del	p.Tyr867Thrfs*47	Unknown	Frameshift	g.157469804del	ARID1B-CSS	(Santen et al., 2013)
54	9	c.2692C>T	p.Arg898*	De novo	Nonsense	g.157469898C>T	ARID1B-CSS	(Wieczorek et al., 2013)
125	9	c.2692C>T	p.Arg898*	De novo	Nonsense	g.157469898C>T	ARID1B-ID	
55	9	c.2723delC	p.Pro908Hisfs*6	De novo	Frameshift	g.157469929del	ARID1B-CSS	(Wieczorek et al., 2013)
32	9	c.2803dup	p.Met935Asnfs*7	Unknown	Frameshift	g.157470009dup	ARID1B-CSS	(Santen et al., 2013)
44	9	c.2877del	p.Ser959Argfs*9	De novo	Frameshift	g.157470083del	ARID1B-CSS	(Santen et al., 2013)
74	10	c.2891_2892insAC	p.Phe964Leufs*5	De novo	Frameshift	g.157488185_157488186insAC	ARID1B-CSS	(Tsurusaki et al., 2014)
96	10	c.2917dup	p.Met973Asnfs*16	De novo	Frameshift	g.157488211dup	ARID1B-ID	
128	10	c.2917dup	p.Met973Asnfs*16	Unknown	Frameshift	g.157488211dup	ARID1B-ID	
41	10	c.2998del	p.Ala1000Argfs*5	De novo	Frameshift	g.157488292del	ARID1B-CSS	(Santen et al., 2013)
47	10	c.2998del	p.Ala1000Argfs*5	De novo	Frameshift	g.157488292del	ARID1B-CSS	
78	11	c.3041del	p.Ala1014Glufs*3	De novo	Frameshift	g.157495157del	ARID1B-ID	
24	11	c.3135+1G>C	r.spl	Unknown	Splice site	g.157495252G>C	ARID1B-CSS	(Santen et al., 2013)
100	12	c.3136-2A>G	r.spl	De novo	Splice site	g.157502101A>G	ARID1B-ID	
81	12	c.3158del	p.Thr1053Metfs*77	De novo	Frameshift	g.157502125del	ARID1B-ID	
37	12	c.3223C>T	p.Arg1075*	De novo	Nonsense	g.157502190C>T	ARID1B-CSS	(Santen et al., 2013)
126	12	c.3223C>T	p.Arg1075*	Unknown	Nonsense	g.157502190C>T	ARID1B-ID	
12	12	c.3304C>T	p.Arg1102*	De novo	Nonsense	g.157502271C>T	ARID1B-ID	
16	12	c.3304C>T	p.Arg1102*	De novo	Nonsense	g.157502271C>T	ARID1B-CSS	(Tsurusaki et al., 2012)
120	12	c.3430C>T	p.Gln1144*	Unknown	Nonsense	g.157505449C>T	ARID1B-ID	(Wieczorek et al., 2013)
134	13	c.(3345+1_3346-1)_ (3550+1_3551-1)	p.0	Unknown	Deletion	_	ARID1B-CSS	
103	13	c.3433_3434insTA	p.Tyr1145Leufs*67	De novo	Frameshift	g.157505452_157505453insTA	ARID1B-ID	(Sonmez et al., 2016)
114	13	c.3473_3515del	p.Arg1158Profs*39	De novo	Frameshift	g.157505492_157505534del	ARID1B-ID	
109	14	c.3568C>T	p.Gln1190*	De novo	Nonsense	g.157510793C>T	ARID1B-CSS	
19	14	c.3586dup	p.Gln1196Profs*14	De novo	Frameshift	g.157510811dup	ARID1B-ID	
121	14	c.3586dup	p.Gln1196Profs*14	De novo	Frameshift	g.157510811dup	ARID1B-ID	(Wieczorek et al., 2013)

94	14	c.3656dup	p.His1220Serfs*26	De novo	Frameshift	g.157510881dup	ARID1B-CSS	
1	15	c.3846dup	p.Gly1283Trpfs*38	De novo	Frameshift	g.157511328dup	ARID1B-CSS	(Santen et al., 2013)
25	16	c.4009C>T	p.Arg1337*	De novo	Nonsense	g.157517445C>T	ARID1B-CSS	(Santen et al., 2013)
73	16	c.4009C>T	p.Arg1337*	Unknown	Nonsense	g.157517445C>T	ARID1B-CSS	(Tsurusaki et al., 2014)
145	16	c.4009C>T	p.Arg1337*	De novo	Nonsense	g.157517445C>T	ARID1B-ID	
3	17	c.4098C>G	p.Tyr1366*	De novo	Nonsense	g.157520029C>G	ARID1B-CSS	(Baban et al., 2008)
87	17	c.4110G>A	r.spl	De novo	Splice site	g.157520041G>A	ARID1B-CSS	
147	17	c.4110+1G>A	r.spl	De novo	Splice site	g.157520042G>A	ARID1B-ID	
56	18	c.4216_4217ins19	p.Gln1406Leufs*59	De novo	Frameshift	g.157521944_157521945ins19	ARID1B-CSS	(Wieczorek et al., 2013)
116	18	c.4304dup	p.Gln1437Alafs*22	Unknown	Frameshift	g.157522032dup	ARID1B-CSS	
34	18	c.4448_4449ins14	p.Pro1489Leufs*10	Unknown	Frameshift	g.157522176_157522177ins14	ARID1B-CSS	(Santen et al., 2013)
66	18	c.4456C>T	p.Gln1486*	De novo	Nonsense	g.157522184C>T	ARID1B-ID	
118	18	c.4566T>A	p.Tyr1522*	De novo	Nonsense	g.157522294T>A	ARID1B-ID	
40	18	c.4620C>A	p.Tyr1540*	De novo	Nonsense	g.157522348C>A	ARID1B-CSS	(Santen et al., 2013)
49	18	c.4622_4631del	p.Gln1541Argfs*35	De novo	Frameshift	g.157522350_157522359del	ARID1B-CSS	(Santen et al., 2013)
117	18	c.4678C>T	p.Gln1560*	Unknown	Nonsense	g.157522406C>T	ARID1B-ID	
35	18	c.4741C>T	p.Gln1581*	De novo	Nonsense	g.157522469C>T	ARID1B-ID	
93	18	c.4741C>T	p.Gln1581*	De novo	Nonsense	g.157522469C>T	ARID1B-CSS	
57	18	c.4770_4771delinsG	p.Gln1591Argfs*23	De novo	Frameshift	g.157522498_157522499delinsG	ARID1B-CSS	(Wieczorek et al., 2013)
13	18	c.4771C>T	p.Gln1591*	De novo	Nonsense	g.157522499C>T	ARID1B-CSS	
72	18	c.4821del	p.Pro1609Leufs*5	De novo	Frameshift	g.157522549del	ARID1B-CSS	(Tsurusaki et al., 2014)
30	18	c.4847C>G	p.Ser1616*	De novo	Nonsense	g.157522575C>G	ARID1B-CSS	
99	18	c.4870C>T	p.Arg1624*	De novo	Nonsense	g.157522598C>T	ARID1B-ID	
106	18	c.4870C>T	p. Arg1624*	De novo	Nonsense	g.157522598C>T	ARID1B-ID	
2	19	c.4911_4915del	p.Trp1637Cysfs*6	De novo	Frameshift	g.157525016_157525020del	ARID1B-CSS	(Santen et al., 2013)
8	19	c.4924_4930del	p.Ser1642Asnfs*40	De novo	Frameshift	g.157525029_157525035del	ARID1B-ID	
123	19	c.5025G>A	r.spl	De novo	Splice site	g.157525130G>A	ARID1B-ID	
29	20	c.5025+1G>A	r.spl	Unknown	Splice site	g.157525131G>A	ARID1B-CSS	
58	20	c.5071delC	p.Leu1691*	Unknown	Frameshift	g.157527346del	ARID1B-CSS	(Wieczorek et al., 2013)
130	20	c.5072del	p.Leu1691Argfs*75	De novo	Frameshift	g.157527347del	ARID1B-ID	
46	20	c.5109_5114delinsT	p.Gly1704Profs*7	De novo	Frameshift	g.157527384_157527389delinsT	ARID1B-ID	
28	20	c.5257G>T	p.Glu1753*	De novo	Nonsense	g.157527532G>T	ARID1B-CSS	
91	20	c.5267_5270del	p.Glu1756Alafs*9	De novo	Frameshift	g.157527542_157527545del	ARID1B-CSS	
113	20	c.5267_5270del	p.Glu1756fs*9	De novo	Frameshift	g.157527542_157527545del	ARID1B-ID	
48	20	c.5329A>T	p.Lys1777*	De novo	Nonsense	g.157527604A>T	ARID1B-CSS	(Santen et al., 2013)
18	20	c.5394_5397del	p.Phe1798Leufs*52	De novo	Frameshift	g.157527669_157527672del	ARID1B-ID	
42	20	c.5394_5397del	p.Phe1798Leufs*52	De novo	Frameshift	g.157527669_157527672del	ARID1B-CSS	(Santen et al., 2013)
90	20	c.5404C>T	p.Arg1802*	De novo	Nonsense	g.157527679C>T	ARID1B-ID	
138	20	c.5404C>T	p.Arg1802*	De novo	Nonsense	g.157527679C>T	ARID1B-ID	
59	20	c.5457G>A	p.Trp1819*	Unknown	Nonsense	g.157527732G>A	ARID1B-CSS	(Wieczorek et al., 2013)
38	20	c.5571_5572del	p.Glu1858Alafs*8	De novo	Frameshift	g.157527848_157527849del	ARID1B-ID	
92	20	c.5594_5595del	p.Ser1865*	De novo	Frameshift	g.157527869_157527870del	ARID1B-ID	

67	20	c.5623_5625delins TGACGTCT	p.Ala1875*	Unknown	Frameshift	g.157527898_157527900delins TGACGTCT	ARID1B-CSS	
136	20	c.5626dup	p.Thr1876Asnfs*4	De novo	Frameshift	g.157527901dup	ARID1B-ID	
17	20	c.5632del	p.Asp1878Metfs*96	Unknown	Frameshift	g.157527907del	ARID1B-CSS	(Tsurusaki et al., 2012)
11	20	c.5635delG	p.Asp1879Thrfs*95	De novo	Frameshift	g.157527910del	ARID1B-CSS	(Santen et al., 2013)
26	20	c.5968C>T	p.Arg1990*	Unknown	Nonsense	g.157528243C>T	ARID1B-CSS	(Santen et al., 2013)
33	20	c.5968C>T	p.Arg1990*	Unknown	Nonsense	g.157528243C>T	ARID1B-CSS	(Santen et al., 2013)
45	20	c.5968C>T	p.Arg1990*	De novo	Nonsense	g.157528243C>T	ARID1B-CSS	(Santen et al., 2013)
112	20	c.5995del	p.Glu1999Argfs*22	De novo	Frameshift	g.157528270del	ARID1B-CSS	
10	20	c.6038G>A	p.Trp2013*	De novo	Nonsense	g.157528313G>A	ARID1B-CSS	(Santen et al., 2013)
60	20	c.6041G>A	p.Trp2014*	Unknown	Nonsense	g.157528316G>A	ARID1B-CSS	(Wieczorek et al., 2013)
61	20	c.6041G>A	p.Trp2014*	Unknown	Nonsense	g.157528316G>A	ARID1B-CSS	(Wieczorek et al., 2013)
69	20	c.6120C>G	p.Tyr2040*	Unknown	Nonsense	g.157528395C>G	ARID1B-CSS	(Tsurusaki et al., 2014)
83	20	c.6198del	p.Pro2068Glnfs*31	De novo	Frameshift	g.157528473del	ARID1B-ID	
27	20	c.6233del	p.Pro2078Leufs*21	De novo	Frameshift	g.157528508del	ARID1B-CSS	(Santen et al., 2013)
105	20	c.6238A>T	p.Arg2080*	De novo	Nonsense	g.157528513A>T	ARID1B-ID	
110	20	c.6250G>T	p.Glu2084*	Unknown	Nonsense	g.157528525G>T	ARID1B-ID	
115	20	c.6347_6350del	p.Val2116Glyfs*28	De novo	Frameshift	g.157528622_157528625del	ARID1B-CSS	
22	20	c.6382C>T	p.Arg2128*	De novo	Nonsense	g.157528657C>T	ARID1B-CSS	(Tsurusaki et al., 2014)
62	20	c.6382C>T	p.Arg2128*	De novo	Nonsense	g.157528657C>T	ARID1B-CSS	(Wieczorek et al., 2013)
111	20	c.6383del	p.Arg2128Glnfs*17	De novo	Frameshift	g.157528658del	ARID1B-ID	
63	20	c.6439dupA	p.Arg2147Lysfs*45	De novo	Frameshift	g.157528714dup	ARID1B-CSS	(Wieczorek et al., 2013)
79	20	c.6490G>T	p.Glu2164*	De novo	Nonsense	g.157528765G>T	ARID1B-ID	
132	20	c.6511C>T	p.Gln2171*	Unknown	Nonsense	g.157528786C>T	ARID1B-ID	
70	20	c.6516C>G	p.Tyr2172*	De novo	Nonsense	g.157528791C>G	ARID1B-CSS	(Tsurusaki et al., 2014)
108	20	c.6644del	p.Phe2215Serfs*17	De novo	Frameshift	g.157528919del	ARID1B-ID	

Used transcript: NM_020732.3. All variants have been uploaded in the LOVD database: www.lovd.nl/ARID1B

Baban A, Moresco L, Divizia MT, et al. Pituitary hypoplasia and growth hormone deficiency in Coffin-Siris syndrome. *Am J Med Genet A.* 2008;146A(3): 384-388. doi:10.1002/ajmg.a.32111

Santen, G. W., Aten, E., Vulto-van Silfhout, A. T., Pottinger, C., van Bon, B. W., van Minderhout, I. J., . . . van Belzen, M. J. (2013). Coffin-Siris syndrome and the BAF complex: genotype-phenotype study in 63 patients. Hum Mutat, 34(11), 1519-1528. doi:10.1002/humu.22394

Sonmez, F. M., Uctepe, E., Gunduz, M., Gormez, Z., Erpolat, S., Oznur, M., . . . Gunduz, E. (2016). Coffin-Siris syndrome with cafe-au-lait spots, obesity and hyperinsulinism caused by a mutation in the ARID1B gene. Intractable Rare Dis Res, 5(3), 222-226. doi:10.5582/irdr.2014.01040

Tsurusaki, Y., Okamoto, N., Ohashi, H., Kosho, T., Imai, Y., Hibi-Ko, Y., . . . Matsumoto, N. (2012). Mutations affecting components of the SWI/SNF complex cause Coffin-Siris syndrome. Nat Genet, 44(4), 376-378. doi:10.1038/ng.2219

Tsurusaki, Y., Okamoto, N., Ohashi, H., Mizuno, S., Matsumoto, N., Makita, Y., . . . Matsumoto, N. (2014). Coffin-Siris syndrome is a SWI/SNF complex disorder. Clin Genet, 85(6), 548-554. doi:10.1111/cge.12225

Wieczorek, D., Bogershausen, N., Beleggia, F., Steiner-Haldenstatt, S., Pohl, E., Li, Y., . . . Wollnik, B. (2013). A comprehensive molecular study on Coffin-Siris and Nicolaides-Baraitser syndromes identifies a broad molecular and clinical spectrum converging on altered chromatin remodeling. Hum Mol Genet, 22(25), 5121-5135. doi:10.1093/hmg/ddt366

Table S2 Extended overview of clinical characteristics of ARID1B patients

				ARID1B-CSS			ARID1B-ID				
Clinical features +	*n* = 143	pt affected	%	*n* = 79	pt affected	%	*n* = 64	pt affected	%	*p* -value	Test
Sex (female)	143	69	48.3%	79	45	57.0%	64	24	37.5%	0.028	*
If the patient was not classified as CSS, does the patient retrospectively fit the CSS spectrum?	59	54	91.5%	-	-	-	59	54	91.5%	-	
Growth parameters & development											
Gestational age, weeks (mean; SD)	133	39.0	2.1	75	39.1	2.0	58	38.9	2.4	0.879	#
Birthweight (<-2 SDS)	129	7	5.4%	74	5	6.8%	55	2	3.6%	0.506	**
Height at birth (<-2 SDS)	43	4	9.3%	27	3	11.1%	16	1	6.3%	0.660	**
OFC at birth (<-2 SDS)	51	2	3.9%	35	1	2.9%	16	1	6.3%	0.232	**
Age last measurements, years (n; median; min-max)	143	10	0-51	79	10	0-36	64	9	0.5-51	0.682	#
Weight (<-2 SDS)	92	6	6.5%	46	4	8.7%	46	2	4.3%	0.571	**
Height (<-2 SDS)	122	37	30.3%	70	26	37.1%	52	11	21.2%	0.177	**
OFC (<-2 SDS)	105	3	2.9%	63	2	3.2%	42	1	2.4%	0.670	**
Motor skills gross, delayed	103	102	99.0%	46	45	97.8%	57	57	100.0%	0.447	**
Motor skills fine, delayed	100	95	95.0%	44	43	97.7%	56	52	92.9%	0.381	**
Sitting,months (mean,SD)	87	13.9	8.30	42	13.1	4.46	45	14.6	10.7	0.884	#
Walking independently, months (mean, SD)	117	28.2	10.5	65	28.1	8.4	52	28.4	12.7	0.238	#
First words, months (mean, SD)	84	40.6	23.6	42	40.6	20.1	42	40.7	26.9	0.673	#
Speech, delayed	131	86	65.6%	75	51	68.0%	56	35	62.5%	0.106	**
No speech		42	32.1%		23	30.7%		19	33.9%		
Obstructive sleep apnea++	71	6	8.5%	34	0	0.0%	37	6	16.2%	0.026	**
Laryngomalacia++	91	18	19.8%	47	8	17.0%	44	10	22.7%	0.466	*
Feeding difficulties	121	84	69.4%	62	39	62.9%	59	45	76.3%	0.111	*
Start of feeding difficulties	71			34			37			0.345	**
Birth		54	76.1%		26	76.5%		28	75.7%		
Before 6 months		12	16.9%		7	20.6%		5	13.5%		
After 6 months		5	7.0%		1	2.9%		4	10.8%		
Duration of feeding problems	58			23			35			0.639	**
Brief		27	46.6%		9	39.1%		18	51.4%		
Several years		4	6.9%		2	8.7%		2	5.7%		
Ongoing		27	46.6%		12	52.2%		15	42.9%		
Tube feeding	65	11	16.9%	22	3	13.6%	43	8	18.6%	0.409	**
0-6 months		7	10.8%		1	4.5%		6	14.0%		
6-12 months		2	3.1%		1	4.5%		1	2.3%		
1-3 years		1	1.5%		0	0.0%		1	2.3%		
Recurrent infections	75	43	57.3%	30	19	63.3%	45	24	53.3%	0.391	*
Upper airway tract		13	17.3%		3	10.0%		10	22.2%		
Lower airway tract		2	2.7%		0	0.0%		2	4.4%		
ENT infections		9	12.0%		3	10.0%		6	13.3%		
Otitis media		11	14.7%		4	13.3%		7	15.6%		
Urinary tract		2	2.7%		1	3.3%		1	2.2%		
Neurological features											
IQ (median; min-max)	35	55.0	26-114	17	48.0	26-114	18	57.0	29-109	0.067	#
Intellectual disability	127	126	99.2%	70	69	98.6%	57	57	100.0%	0.015	**
Normal-mild		4	3.1%		1	1.4%		3	5.3%		
Mild		36	28.3%		27	38.6%		9	15.8%		
Mild-moderate		20	15.7%		6	8.6%		14	24.6%		
Moderate		28	22.0%		16	22.9%		12	21.1%		
Moderate-severe		21	16.5%		12	17.1%		9	15.8%		
Severe		17	13.4%		7	10.0%		10	17.5%		
Hypotonia	116	94	81.0%	71	57	80.3%	45	37	82.2%	0.795	*
Seizures	142	39	27.5%	78	22	28.2%	64	17	26.6%	0.880	**
No seizures, but abnormal EEG		8	5.6%		5	6.4%		3	4.7%		
Seizure start year (median, SD)	37	4.0	4.0	21	5.0	3.3	16	2.5	4.8	0.644	#
Seizure types	23			10			13			0.953	*
Absence seizures (petit mal seizures)		2	8.7%		1	10.0%		1	7.7%		
Myoclonic seizures		2	8.7%		1	10.0%		1	7.7%		
Tonic seizures		2	8.7%		1	10.0%		1	7.7%		
Tonic-clonic seizures		11	47.8%		4	40.0%		7	53.8%		
Atonic seizures		1	4.3%		1	10.0%		0	0.0%		
Focal seizures		2	8.7%		1	10.0%		1	7.7%		
Febrile seizures	93	3	3.2%	53	2	3.8%	40	1	2.5%	1.000	**
Seizure frequency	18			9			9			0.671	**
Once		5	27.8%		1	11.1%		4	44.4%		
Less than once a year		2	11.1%		2	22.2%		0	0.0%		
Once a year		6	33.3%		4	44.4%		2	22.2%		
Once a month		2	11.1%		1	11.1%		1	11.1%		
1/2 a month		1	5.6%		0	0.0%		1	11.1%		
≥ 2 per month		1	5.6%		0	0.0%		1	11.1%		
Agenesis of the corpus callosum	101	29	28.7%	62	18	29.0%	39	11	28.2%	0.344	**
Partial/hypoplasia		14	13.9%		11	17.7%		3	7.7%		
Neuroradiology	47	41	87.2%	17	16	94.1%	30	25	83.3%	0.305	*
Delayed myelination		8	17.0%		2	11.8%		6	20.0%		
Mega cisterna magna		7	14.9%		4	23.5%		3	10.0%		
Colpocephaly		5	10.6%		2	11.8%		3	10.0%		
Hypoplastic splenium		1	2.1%		0	0.0%		1	3.3%		
Hypoplasia		2	4.3%		0	0.0%		2	6.7%		
Hyperintense focal lesions		1	2.1%		0	0.0%		1	3.3%		
Enlarged Virchow-Robin spaces		2	4.3%		1	5.9%		1	3.3%		
Pachygyria		1	2.1%		1	5.9%		0	0.0%		
Hippocampal malrotation		1	2.1%		1	5.9%		0	0.0%		
Interhemispheric cyst		1	2.1%		1	5.9%		0	0.0%		
Vision and hearing impairments											
Vision impaired	109	53	48.6%	62	28	45.2%	47	25	53.2%	0.406	*
Vision problems	68	48	70.6%	33	26	78.8%	35	22	62.9%	0.320	**

Astigmatism		11	16.2%		8	24.2%		3	8.6%		
Strabismus		21	30.9%		12	36.4%		9	25.7%		
Optic nerve hypoplasia		2	2.9%		2	6.1%		0	0.0%		
Nystagmus		6	8.8%		2	6.1%		4	11.4%		
Refraction error		7	10.3%		3	9.1%		4	11.4%		
Myopia	102	28	27.5%	59	11	18.6%	43	17	39.5%	0.020	*
Hypermetropia	50	9	18.0%	21	6	28.6%	29	3	10.3%	0.140	**
Abnormal eye exam	40	7	17.5%	15	1	6.7%	25	6	24.0%	0.224	**
Hearing loss	122	27	22.1%	71	13	18.3%	51	14	27.5%	0.157	*
Hearing loss, conductive		8	6.6%		1	1.4%		7	13.7%		
Hearing loss. bilateral		14	11.5%		6	8.5%		8	15.7%		
Hearing loss, unilateral		6	4.9%		4	5.6%		2	3.9%		
Eartubes	122	6	4.9%	71	3	4.2%	51	3	5.9%		
Start hearing problems, congenital	11	7	63.6%	3	2	66.7%	8	5	62.5%	0.109	**
Hearing aid	5	4	80.0%	2	2	100.0%	3	2	66.7%	0.665	**
Dysmorphic features											
Coarse face	121	99	81.8%	62	56	90.3%	59	43	72.9%	0.013	*
Hairline (low anterior and/or posterior)	91	63	69.2%	45	34	75.6%	46	29	63.0%	0.196	*
Scalp hair, abnormal	129	102	79.1%	78	65	83.3%	51	37	72.5%	0.141	*
Sparse		75	58.1%		49	62.8%		26	51.0%		
Forehead (broad or narrow)	95	40	42.1%	49	14	28.6%	46	26	56.5%	0.000	**
Broad		21	22.1%		3	6.1%		18	39.1%		
Narrow		19	20.0%		11	22.4%		8	17.4%		
Eyelashes, long	131	83	63.4%	79	60	75.9%	52	23	44.2%	0.000	*
Eyebrows, thick	134	109	81.3%	78	71	91.0%	56	38	67.9%	0.001	*
Ptosis	133	27	20.3%	77	16	20.8%	56	11	19.6%	0.872	*
Tear duct non-functioning or absent	93	14	15.1%	54	9	16.7%	39	5	12.8%	0.609	*
Nasal bridge, abnormal	100	61	61.0%	59	37	62.7%	41	24	58.5%	0.050	*
Wide		34	34.0%		24	40.7%		10	24.4%		
Flat		21	21.0%		12	20.3%		9	22.0%		
Broad		12	12.0%		3	5.1%		9	22.0%		
Nasal tip, abnormal	129			76			53			0.002	*
Broad		75	58.1%		47	61.8%		28	52.8%		
Upturned (anteverted nares)		38	29.5%		30	39.5%		8	15.1%		
Nose, abnormal	83	39	47.0%	43	23	53.5%	40	16	40.0%	0.022	*
Short		22	26.5%		17	39.5%		5	12.5%		
Long		17	20.5%		6	14.0%		11	27.5%		
Alae nasi, thick	107	59	55.1%	69	46	66.7%	38	13	34.2%	0.001	*
Nasal base, broad	88	43	48.9%	48	21	43.8%	40	22	55.0%	0.392	*
Philtrum, abnormal	109	86	78.9%	72	62	86.1%	37	24	64.9%	0.001	*
Short		27	24.8%		21	29.2%		6	16.2%		
Long		48	44.0%		35	48.6%		13	35.1%		
Broad		38	34.9%		32	44.4%		6	16.2%		
Mouth, large	131	90	68.7%	75	57	76.0%	56	33	58.9%	0.037	*
Upper vermillion, abnormal	127	72	56.7%	75	45	60.0%	52	27	51.9%	0.366	*
Thin		45	35.4%		34	45.3%		11	21.2%		
Thick		27	21.3%		11	14.7%		16	30.8%		
Lower vermillion, thick	125	87	69.6%	76	60	78.9%	49	27	55.1%	0.005	*
Lower lip, drooping	71	40	56.3%	30	23	76.7%	41	17	41.5%	0.004	*
Cleft palate/submucous cleft	90	6	6.7%	35	5	14.3%	55	1	1.8%	0.031	*
Cleft palate		2	2.2%		2	5.7%		0	0.0%		
Cleft uvula		1	1.1%		1	2.9%		0	0.0%		
Bifid uvula		2	2.2%		2	5.7%		0	0.0%		
Submucous cleft		3	3.3%		2	5.7%		1	1.8%		
High arched palate	85	14	16.5%	31	7	22.6%	54	7	13.0%	0.250	*
Ears, abnormal	122	64	52.5%	66	38	57.6%	56	26	46.4%	0.433	*
Low-set		12	9.8%		9	13.6%		3	5.4%		
Posterior rotated		9	7.4%		6	9.1%		3	5.4%		
Hypertrichosis	128	111	86.7%	76	72	94.7%	52	39	75.0%	0.001	*
Musculoskeletal anomalies											
Scoliosis	123	32	26.0%	70	19	27.1%	53	13	24.5%	0.743	*
Pectus, excavatum	104	14	13.5%	57	8	14.0%	47	6	12.8%	0.850	*
Primary dentition, delayed	65	29	44.6%	40	20	50.0%	25	9	36.0%	0.313	*
Permanent dentition, delayed	33	16	48.5%	18	6	33.3%	15	10	66.7%	0.056	*
Widely spaced teeth	72	30	41.7%	40	16	40.0%	32	14	43.8%	0.748	*
Bone age, delayed	40	19	47.5%	30	14	46.7%	10	5	50.0%	1.000	**
Joint laxity	88	53	60.2%	52	32	61.5%	36	21	58.3%	0.763	*
Early arthritis	75	4	5.3%	36	2	5.6%	39	2	5.1%	1.000	**
Clinodactyly	77	28	36.4%	42	19	45.2%	35	9	25.7%	0.076	*
Short phalanges	49	17	34.7%	34	14	41.2%	15	3	20.0%	0.151	*
Complete absent or small 5th distal phalanx	110	44	40.0%	66	40	60.6%	44	4	9.1%	0.000	**
Prominent distal phalanges;	102	25	24.5%	64	20	31.3%	38	5	13.2%	0.040	*
Prominent interphalangeal joints;	103	22	21.4%	64	18	28.1%	39	4	10.3%	0.032	*
Brachydactyly general	60	10	16.7%	19	3	15.8%	41	7	17.1%	1.000	**
Brachydactyly fifth finger	68	21	30.9%	22	11	50.0%	46	10	21.7%	0.018	*
Small nails	122	67	54.9%	73	50	68.5%	49	17	34.7%	0.000	*
Which nails, 5th finger and/or toe	106	59	55.7%	67	44	65.7%	39	15	38.5%	0.007	*
Which nails, all	53	6	11.3%	29	6	20.7%	24	0	0.0%	0.027	**
Fetal finger pads	100	29	29.0%	50	13	26.0%	50	16	32.0%	0.509	*
Intestinal											
Inguinal hernia	90	7	7.8%	46	1	2.2%	44	6	13.6%	0.056	**
Intestinal problems	105	51	48.6%	60	22	36.7%	45	29	64.4%	0.000	*
Constipation		32	30.5%		13	21.7%		19	42.2%		
Gastroesophageal reflux		18	17.1%		8	13.3%		10	22.2%		
Diarrhea		5	4.8%		2	3.3%		3	6.7%		
Pyloric Stenosis		3	2.9%		3	5.0%		0	0.0%		
Umbilical hernia		5	4.8%		1	1.7%		4	8.9%		
Cardiac & renal anomalies											
Cardiac anomalies	113	22	19.5%	69	15	21.7%	44	7	15.9%	0.492	*
ASD		12	10.6%		9	13.0%		3	6.8%		
VSD		6	5.3%		4	5.8%		2	4.5%		

AVSD		1	0.9%		1	1.4%		0	0.0%		
Abnormal aortic arch		3	2.7%		1	1.4%		2	4.5%		
Aortic valve abnormality		3	2.7%		2	2.9%		1	2.3%		
Mitralis insufficiency		3	2.7%		3	4.3%		0	0.0%		
Tricuspid insufficiency		1	0.9%		0	0.0%		1	2.3%		
AV-block		1	0.9%		1	1.4%		0	0.0%		
Patent ductus arteriosus		1	0.9%		0	0.0%		1	2.3%		
Patent foramen ovale		1	0.9%		1	1.4%		0	0.0%		
Enlarged right venticle		1	0.9%		1	1.4%		0	0.0%		
Renal anomalies	95	12	12.6%	53	6	11.3%	42	6	14.3%	0.666	**
Hydronephrotic kidney		3	3.2%		1	1.9%		2	4.8%		
Unilateral ptosis kidney		1	1.1%		0	0.0%		1	2.4%		
Renal failure - Grade IV		1	1.1%		0	0.0%		1	2.4%		
Nephrolithiasis		3	3.2%		2	3.8%		1	2.4%		
Secondary left-sided reflux; left atropic kidney		1	1.1%		0	0.0%		1	2.4%		
Renal abcesses		1	1.1%		1	1.9%		0	0.0%		
Renal sonography, abnormal	43	11	25.6%	19	4	21.1%	24	7	29.2%	0.105	**
Grade 1 increased echo		1			0			1			
Dilated right pyelum		1			0			1			
Cryptorchidism	65	36	55.4%	28	11	39.3%	37	25	67.6%	0.023	*
Endocrinological abnormalities++											
Diabetes mellitus	71	5	7.0%	43	3	7.0%	28	2	7.1%	1.000	**
Type 2 diabetes mellitus	4	3	75.0%	2	2	100.0%	2	1	50.0%	1.000	**
Age (years) diagnosis (nr, median, min-max)	2	32	18,46	1	18.0	18.0	1	46.0	46.0	0.317	#
Hypothyroidism	63	12	19.0%	38	6	15.8%	25	6	24.0%	0.417	*
Age (years) diagnosis (nr, median, min-max)	10	8	1-40	4	2.5	1.3-36.0	6	12.5	1.0-40.0	0.394	#
Growth hormone deficiency	51	7	13.7%	33	6	18.2%	18	1	5.6%	0.398	**
Growth hormone supplementation	50	6	12.0%	31	5	16.1%	19	1	5.3%	0.387	**
Behavioral abnormalities	71	59	83.1%	28	24	85.7%	43	35	81.4%	0.945	*
Hyperkinetic		11	15.5%		4	14.3%		7	16.3%		
Short attention		18	25.4%		7	25.0%		11	25.6%		
Impulsiveness		10	14.1%		4	14.3%		6	14.0%		
Obsessive		11	15.5%		4	14.3%		7	16.3%		
Rigid		6	8.5%		1	3.6%		5	11.6%		
Anger outbursts		12	16.9%		3	10.7%		9	20.9%		
Aggressive		12	16.9%		4	14.3%		8	18.6%		
Anxious		17	23.9%		5	17.9%		12	27.9%		
Poor sociability		14	19.7%		5	17.9%		9	20.9%		
Hyperactivity	63	27	42.9%	25	15	60.0%	38	12	31.6%	0.026	*
High pain threshold++	47	19	40.4%	28	8	28.6%	19	11	57.9%	0.044	*
Psychiatric disorders											
ADHD	48	16	33.3%	16	8	50.0%	32	8	25.0%	0.083	*
Autistic traits	77	44	57.1%	27	18	66.7%	50	26	52.0%	0.215	*
Malignancies	97	1	1.0%	53	0	0.0%	44	1	2.3%	0.454	**
Technique	143			79			64			0.000	**
Sanger sequencing		16	11.2%		7	8.9%		9	14.1%		
Whole exome sequencing		43	30.1%		5	6.3%		38	59.4%		
Targeted NGS < 10 genes		60	42.0%		60	75.9%		0	0.0%		
Targeted NGS < 100 genes		4	2.8%		1	1.3%		3	4.7%		
Targeted NGS > 100 genes		7	4.9%		2	2.5%		5	7.8%		
Genome-wide CNV analysis e.g. microarray		13	9.1%		4	5.1%		9	14.1%		

* Chi-square, ** Fisher's, # Mann-Whitney U

+ the total number of a feature can differ from the sum of subcategories, because in some cases it was possible to answer with more than 1 option or to report the existence of a feature without specifying.

++ data regarding these features were collected through e-mail after first analyses

Abbreviations

ARID1B-CSS: patient group with a suspicion of Coffin-Siris Syndrome before genetic testing
ARID1B-ID: patient group with no suspicion of Coffin-Siris Syndrome before genetic testing
CSS: Coffin-Siris Syndrome
SDS: Standard Deviation Score
OFC: OccipitoFrontal Circumference
SD: Standard Deviation
ENT: Ear Nose Throat
EEG: ElectroEncephaloGraphy
ASD: Atrial Septal Defect
VSD: Ventricular Septal Defect
AV-Block: AtrioVentricular Block
AVSD: AtrioVentricular Septal Defect
NGS: Next Generation Sequencing
CNV: Copy Number Variation
ADHD: Attention Deficit Hyperactivity Disorder

Table S3: Suspected diagnoses* before genetic testing

	Fits CSS spectrum?			
n=60	Yes	No	Unknown	Total
Non-syndromic ID	8	3	2	13
Syndromic ID**	34	1	1	36
Multiple congenital anomalies	3	1	0	4
Developmental delay	4	0	0	4
Neurofibromatosis	0	0	1	1
Autism spectrum disorder	3	0	2	5
Rasopathy	0	1	0	1
Trichothiodystrophy	1	0	0	1
Mucopolysaccharidosis	2	0	0	2
Mucolipidosis	1	0	0	1
No diagnosis reported a priori	4	0	0	4
Total	53	6	5	64

***Syndromic ID specified*

	Fits CSS spectrum?			
n=36	Yes	No	Unknown	Total
Nicolaides-Baraitser syndrome	4	0	0	4
Pitt Hopkins	2	0	0	2
Costello syndrome	2	0	0	2
Coffin-Lowry syndrome	2	0	0	2
Pierpont syndrome	1	0	0	1
Rubinstein-Taybi syndrome	0	0	1	1
Borjeson-Forssman-Lehmann syndrome	0	1	0	1
Wiedemann-Steiner syndrome	1	0	0	1
Not specified	23	0	0	23
Total	34	1	1	36

**More suspected diagnoses possible per patient*

Table S4: Results 'open-ended' questions. Except for growth hormone deficiency only features reported ≥ 3 times were shown.

Reported features	Results open-ended questions Frequency	Results additional data request *n* = 95	pt affected	%
Laryngomalacia	12	91	18	20%
Hyperhidrosis(excessive sweating)	8	-	-	-
Pes planus/ flatfoot	7	-	-	-
Hypothyroidism	6	63	12	19%
Diabetes	4	71	5	7%
Obstructive sleep apnoea	4	71	6	8%
Excessive salivation/drooling	4	-	-	-
Sleep problems/fatigue	4	-	-	-
High pain threshold	3	47	19	40%
High arched eyebrows	3	-	-	-
Prognathia	3	-	-	-
Deep palmar creases of the hands	3	-	-	-
Thin nails (not small)	3	-	-	-
Growth hormone deficiency	2	51	7	14%
Growth hormone supplementation	1	50	6	12%

Table S5: gnomAD, LoF mutations in transcript ENST00000346085 (NM_020732.3)
http://gnomad.broadinstitute.org/transcript/ENST00000346085

Exon	Transcript Consequence	Location gdna	Predicted protein change	Allele Count	Interpretation
1	c.362_363insGC	g.156778291_156778292insGC	p.Gln122Hisfs*59	39	Begin exon 1 in low complexity regions (LCR)
1	c.363_364insG	g.156778292_156778293insG	p.Gln122Alafs*110	6	Begin exon 1, in LCR
1	c.363_364insGCAG	g.156778292_156778293insGCAG	p.Gln122Alafs*111	4	Begin exon 1, in LCR
3	c.1738-1G>A	g.156871613G>A	r.spl?	1	Exon 3
3	c.1762G>T	g.156871638G>T	p.Glu588*	4	Exon 3
5	c.1927-2A>G	g.156935464A>G	r.spl?	1	Likely pathogenic
11	c.3026-1G>A	g.157174007G>A	r.spl?	3	Likely pathogenic
12	c.3345+2T>C	g.157181180T>C	r.spl?	1	Likely pathogenic
15	c.3780_3817del	g.157190128_157190165del	p.Gln1261Alafs*3	1	Likely pathogenic
20	c.6013delG	g.157207154del	p.Val2005Trpfs*16	1	Likely pathogenic

Table S6: Data DECIPHER database compared with data www.arid1bgene.com

	DECIPHER (*n*=54)			ARID1Bgene.com (*n*=143)				
**Reported (HPO-)feature**	pt affected	%	*p*-value*	*n*	pt affected	%	*p*-value**	**Reported feature**
Intellectual disability	11	20.4	-	127	126	99.2	<0.001	Cognition delay
Morphological abnormality of the gastrointestinal tract	12	22.2	+	105	51	48.6	0.001	Intestinal problems
Agenesis of corpus callosum	10	18.5	+	101	43	42.6	0.003	Agenesis of corpus callosum
Muscular hypotonia	9	16.7	-	116	94	81.0	<0.001	Hypotonia
Hypertrichosis	8	14.8	+	128	111	86.7	<0.001	Hypertrichosis
Short stature	8	14.8	-	122	37	30.3	0.030	Height < -2 SDS
Laryngomalacia	7	13.0	+	91	18	19.8	0.293	Laryngomalacia++
Abnormality of the testis	7	13.0	+	65	36	55.4	<0.001	Cryptorchidism
Absent speech	4	7.4	-	131	42	32.1	<0.001	No speech
Expressive language delay	4	7.4	-	131	128	97.7	<0.001	Speech delay
Myopia	4	7.4	-	102	28	27.5	0.003	Myopia
Abnormality of the cardiac septa	3	5.6	-	106	16	15.1	0.078	VSD/ASD/AVSD
Abnormality of the kidney	2	3.7	-	95	12	12.6	0.073	Renal anomalies
Seizures	3	5.6	-	142	39	27.5	0.001	Seizures

*p-value based on frequency of the reported feature in other phenotyped patients in DECIPHER; ‘+’ is a p-value < 0.05/247
** p-value compared to frequency in DECIPHER cohort
***no question was included
++ data collected through e-mail after first analyses

Figure S1. Photographs of two patients with ARID1B-CSS and two patients with ARID1B-ID. Relatively coarse facial features can be seen in these patients, and most have full lips. Typical sparse hair is seen in patient 142. The extremities show short fifth digit in patient 1, hypoplastic fifth finger nail in patient 37, hypoplastic nail of the fifth toe of patient 132, and normal nails in patient 142. Overall the characteristics represent a spectrum with patients with ARID1B-CSS and ARID1B-ID having overlapping features.

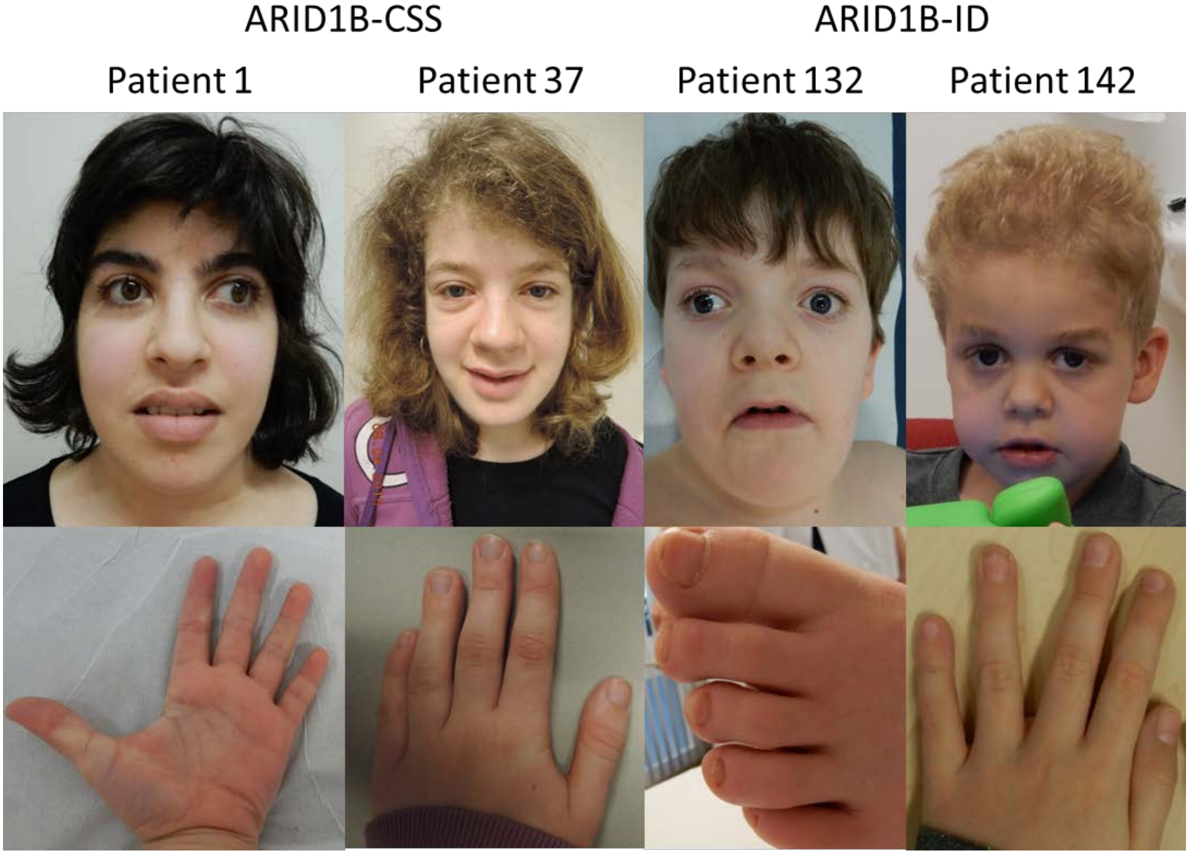

Figure S2: Degree of intellectual disability and mutation position

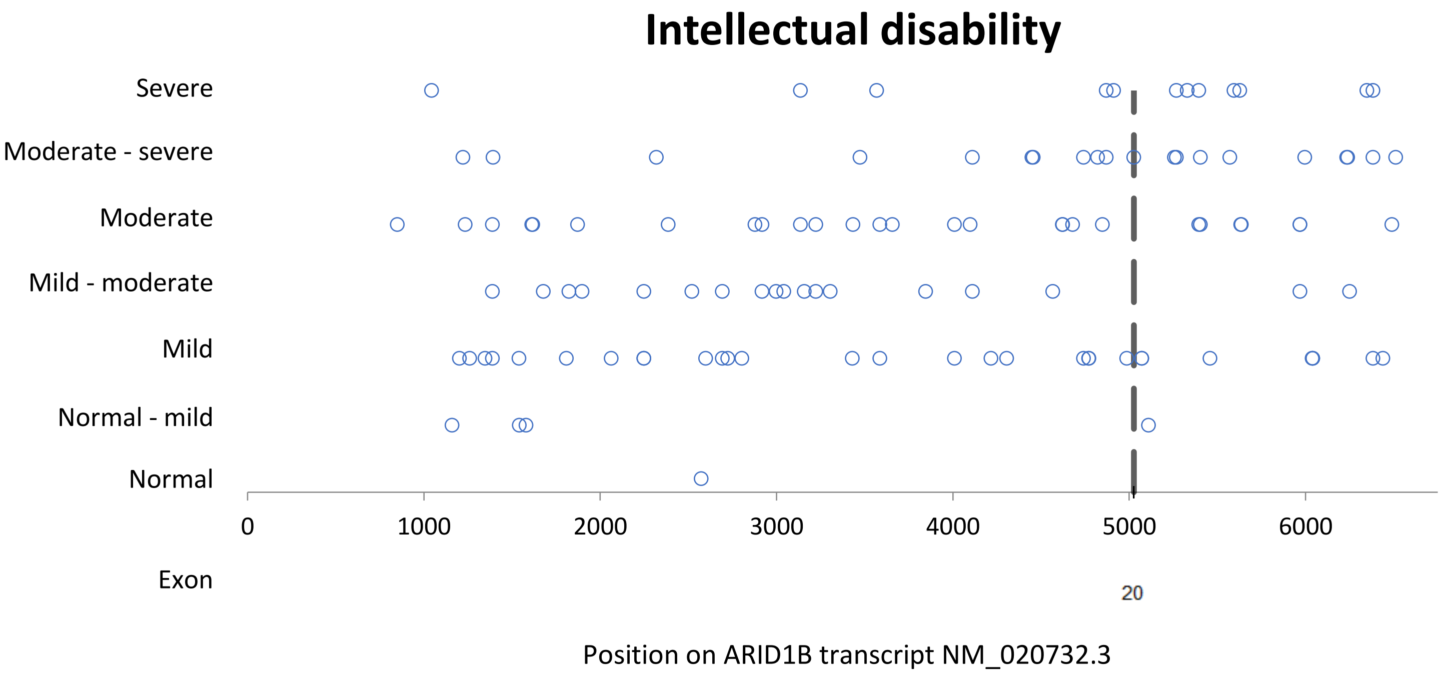

Figure S3: Data DECIPHER database compared with data www.arid1bgene.com

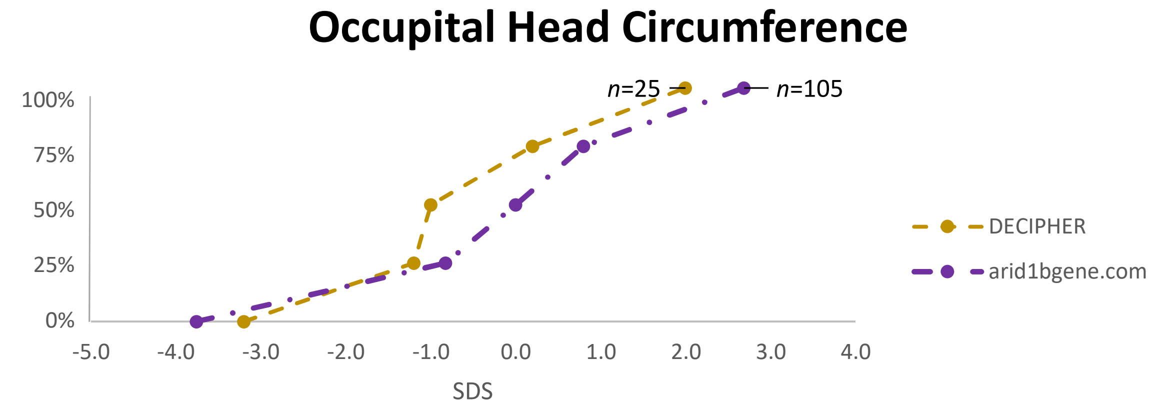

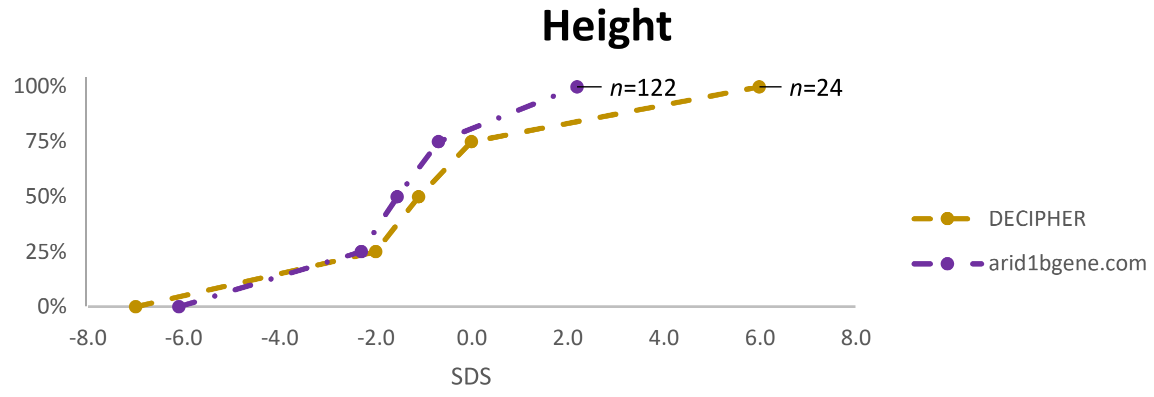

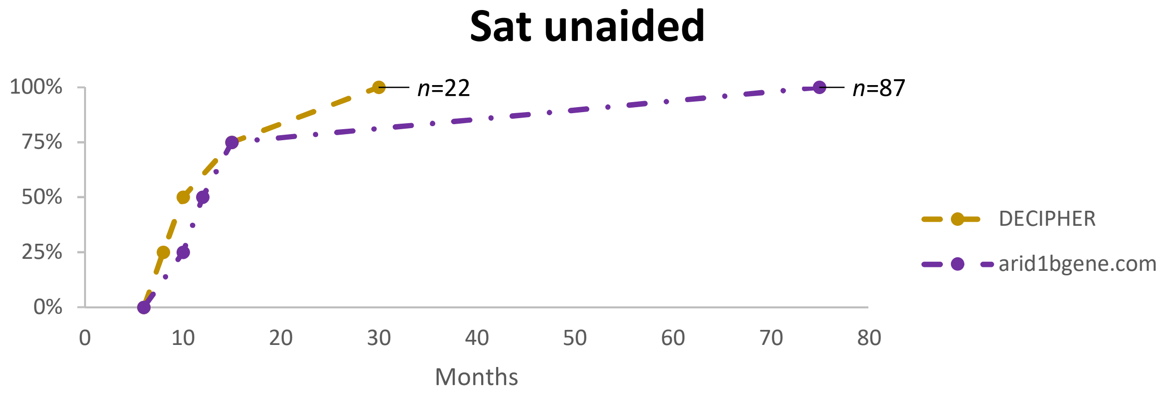

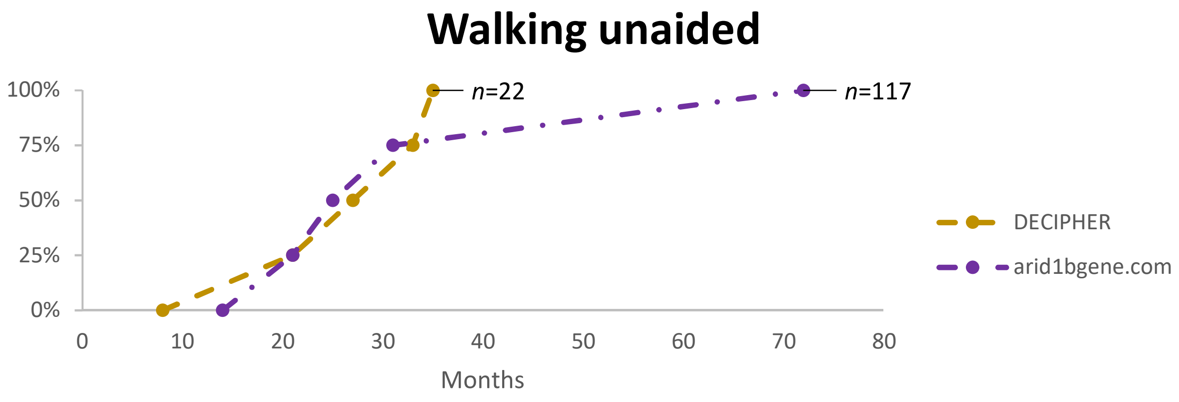

Walking unaided
100%
75%
50%
25%
0%
0
10
20
30
40
50
60
70
80
Months
n=22
n=117
DECIPHER
arid1bgene.com

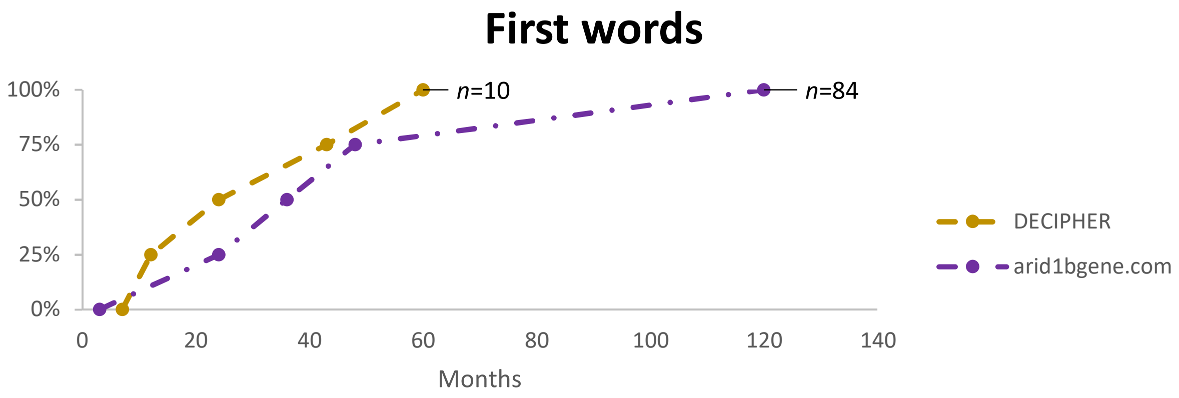

First words
100%
75%
50%
25%
0%
0
20
40
60
80
100
120
140
Months
n=10
n=84
DECIPHER
arid1bgene.com
